# Copper primes adaptation of uropathogenic *Escherichia coli* to superoxide stress by activating superoxide dismutases

**DOI:** 10.1371/journal.ppat.1008856

**Published:** 2020-08-26

**Authors:** Panatda Saenkham, Matthew Ritter, George L. Donati, Sargurunathan Subashchandrabose

**Affiliations:** 1 Department of Veterinary Pathobiology, College of Veterinary Medicine and Biomedical Sciences, Texas A&M University, College Station, Texas, United States of America; 2 Department of Chemistry, Wake Forest University, Winston-Salem, North Carolina, United States of America; University of California Davis School of Medicine, UNITED STATES

## Abstract

Copper and superoxide are used by the phagocytes to kill bacteria. Copper is a host effector encountered by uropathogenic *Escherichia coli* (UPEC) during urinary tract infection in a non-human primate model, and in humans. UPEC is exposed to higher levels of copper in the gut prior to entering the urinary tract. Effects of pre-exposure to copper on bacterial killing by superoxide has not been reported. We hypothesized that copper-replete *E*. *coli* is more sensitive to killing by superoxide *in vitro*, and in activated macrophages. We utilized wild-type UPEC strain CFT073, and its isogenic mutants lacking copper efflux systems, superoxide dismutases (SODs), regulators of a superoxide dismutase, and complemented mutants to address this question. Surprisingly, our results reveal that copper protects UPEC against killing by superoxide *in vitro*. This copper-dependent protection was amplified in the mutants lacking copper efflux systems. Increased levels of copper and manganese were detected in UPEC exposed to sublethal concentration of copper. Copper activated the transcription of *sodA* in a SoxR- and SoxS-dependent manner resulting in enhanced levels of SodA activity. Importantly, pre-exposure to copper increased the survival of UPEC within RAW264.7 and bone marrow-derived murine macrophages. Loss of SodA, but not SodB or SodC, in UPEC obliterated copper-dependent protection from superoxide *in vitro*, and from killing within macrophages. Collectively, our results suggest a model in which sublethal levels of copper trigger the activation of SodA and SodC through independent mechanisms that converge to promote the survival of UPEC from killing by superoxide. A major implication of our findings is that bacteria colonizing copper-rich milieus are primed for efficient detoxification of superoxide.

## Author summary

Copper and superoxide are used by the host immune cells to limit bacterial growth during infection. We investigated the interplay between copper and superoxide in killing uropathogenic *E*. *coli*, the causative agent of urinary tract infection, by utilizing bacteriological, genetic, molecular biology, and cell culture approaches. Our findings reveal that exposure to sublethal levels of copper helps *E*. *coli* to survive better in the presence of superoxide by activating superoxide dismutases. Importantly, pre-exposure to copper protects *E*. *coli* from killing by macrophages, an important subset of immune cells that control bacterial growth during infection. By shedding light on a novel copper-dependent protection from superoxide in *E*. *coli*, findings of this report have major ramifications for bacterial adaptation to survival under superoxide stress.

## Introduction

Bacterial urinary tract infection (UTI) is one the most common infectious conditions encountered by people globally [1]. Children, women, elderly, diabetics, and individuals with uroliths and urinary catheters are at a higher risk for developing UTI [2]. Uropathogenic *Escherichia coli* (UPEC) is the predominant etiological agent of UTI [2–4]. Other pathogens including *Klebsiella pneumoniae*, *Proteus mirabilis*, *Staphylococcus aureus*, and *Enterococcus faecalis* also cause UTI in a significant number of patients [2]. Uropathogens utilize numerous fitness and virulence mechanisms to successfully colonize the urinary tract, and to outmaneuver the effectors of host immunity [3–5]. Human urinary copper and ceruloplasmin, the primary copper-containing protein in mammals, content is elevated during UTI caused by UPEC, *K*. *pneumoniae* and *P*. *mirabilis* [6,7]. Complexes of copper with yersiniabactin, a siderophore, has been detected in the urine of patients with UTI [8]. UPEC-induced UTI also leads to urinary copper mobilization in a non-human primate model [6]. Collectively, these studies highlight an important and novel biological role for copper in host-pathogen interaction during UTI.

Recent evidence indicates that copper exerts its toxicity via interaction with specific targets in *E*. *coli* [9,10]. Historically, copper toxicity was linked with generation of reactive oxygen species (ROS) from participating in Fenton, and Haber-Weiss reactions [11]. Copper-responsive transcriptional changes in *E*. *coli* have revealed activation of the SoxRS regulon [12–14]. Copper remains toxic under anaerobiosis where ROS is not generated, raising questions on the role of ROS in copper toxicity [9,15]. ROS-independent disruption of iron-sulfur clusters in dehydratases involved in the biosynthesis of branched-chain amino acids has emerged as a molecular mechanism of copper toxicity in *E*. *coli* [9]. Copper also decreases the activity of glutamate oxoglutarate aminotransferase, an iron-sulfur cluster-containing enzyme, to induce glutamate starvation in *E*. *coli* [10]. Phagocytes are the primary effectors of innate immunity that hamper bacterial growth during UTI [16]. Copper is found within phagolysosomes of macrophages, in addition to superoxide, hydrogen peroxide, lysozyme and other antibacterial agents [17–20]. Copper and superoxide, a mediator of oxidative stress, exert toxic effects on *E*. *coli* independently [7,9,21]. UPEC colonizes the gut prior to causing UTI where it is exposed to higher levels of copper from dietary sources, and biliary excretion of excess copper, than the urinary tract. However, the impact of pre-exposure to copper on killing by superoxide in bacterial pathogens has not been reported, and is the subject of this investigation.

Central hypothesis that was tested in this study is that copper-replete *E*. *coli* is more sensitive to killing by superoxide *in vitro*, and by activated macrophages. We utilized UPEC mutants defective in copper efflux, lacking superoxide dismutase (SOD) activity, regulators of *sodA* expression, double mutants lacking both, and complemented mutants to test this hypothesis. We determined UPEC viability *in vitro*, metal content, transcript levels, activity of SODs, and killing in macrophages to address the role of copper in modulating sensitivity to superoxide in *E*. *coli*. Evidence presented in this report collectively supports a role for copper in protecting *E*. *coli* from killing by superoxide, and by macrophages in a *sodA*-dependent mechanism.

## Results

### Sensitivity of UPEC copper efflux-defective mutants to copper

We utilized isogenic *ΔcopA*, *Δcus* and *ΔcopAΔcus* copper efflux-defective mutants in UPEC clinical isolate CFT073 (wild-type, Table 1) to test the effect of pre-exposure to copper on superoxide sensitivity. CopA is an inner membrane copper efflux pump, and is the primary copper homeostasis system under aerobic conditions [22]. CusCFBA is a cell envelope-spanning RND-type efflux system that is active under low oxygen conditions, and at extremely high copper stress [15]. Sensitivity of wild-type and copper efflux-defective mutant cells in logarithmic and stationary phases to copper was determined under aerobic condition (S1 Fig). Enumeration of bacterial counts revealed that *ΔcopA* and *ΔcopAΔcus* mutants were more sensitive to copper (3 mM CuSO_4_) than wild-type and *Δcus* mutant strain during stationary phase (S1 Fig). There were no differences in copper sensitivity among these strains at other concentrations, or in cells from logarithmic growth phase (S1 Fig). Our results are consistent with known roles of CopA and CusCFBA in copper homeostasis in *E*. *coli* [15,22]. UPEC resides in the large intestine prior to entering the urinary tract [23,24]. We determined the level of copper in the colon contents of mice by inductively coupled plasma mass spectrometry (ICP-MS). Colon contents of C57BL/6 mice fed a standard laboratory rodent diet contained 22.6 ± 8.3 PPM of copper (S2 Fig). Based on *in vitro* sensitivity (S1 Fig), and copper level in the mouse colon contents, we used 0.5 to 2 mM CuSO_4_ (32 to 128 PPM of copper) to induce sublethal copper stress in subsequent experiments.

**Table 1:** Strains and plasmids used in this study.

Strain	Description^a^	Source
CFT073	Wild-type UPEC	[63]
*ΔcopA*	CFT073 *ΔcopA*::*npt*	A. Hyre
*ΔcusSRCFBA*	CFT073 *ΔcusSRCFBA*::*npt*	[7]
*ΔcopAΔcus*	CFT073 *ΔcopA*::*cat*, *Δcus*::*npt*	A. Hyre
*ΔsodA*	CFT073 *ΔsodA*::*npt*	This study
*ΔsodB*	CFT073 *ΔsodB*::*npt*	This study
*ΔsodC*	CFT073 *ΔsodC*::*npt*	This study
*ΔsoxR*	CFT073 *ΔsoxR*::*npt*	This study
*ΔsoxS*	CFT073 *ΔsoxS*::*npt*	This study
*ΔsodAΔcopA*	CFT073 *ΔsodA*::*npt*, *ΔcopA*::*cat*	This study
*ΔsodBΔcopA*	CFT073 *ΔsodB*::*npt*, *ΔcopA*::*cat*	This study
*ΔsodCΔcopA*	CFT073 *ΔsodC*::*npt*, *ΔcopA*::*cat*	This study
**Plasmids**		
pGen	Low copy number vector	[65]
p*copA*	pGen_*copA*	This study
p*sodA*	pGen_*sodA*	This study

^**a**^UPEC, uropathogenic *E*. *coli*; *npt*, neomycin phosphotransferase; *cat*, chloramphenicol acetyltransferase

### Copper increases resistance to superoxide in UPEC

**Fig 1:**
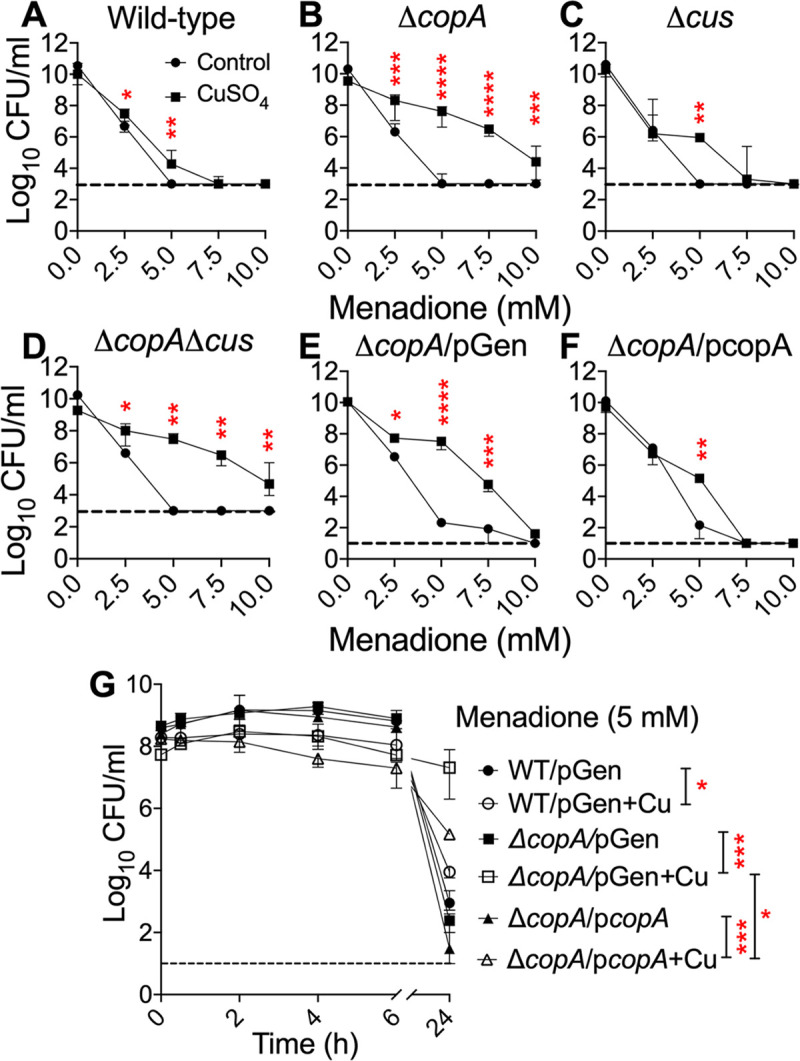
Pre-exposure to copper protects UPEC from killing by superoxide. Wild-type UPEC strain CFT073 (A), *ΔcopA* (B), Δ*cus* (C), *ΔcopAΔcus* (D), *ΔcopA/*pGen (E), and *ΔcopA/*p*copA* (F) strains were grown in LB (circles) or LB with copper (squares) to early logarithmic phase (OD_600_ = 0.3, ~10^8^ CFU/ml). Menadione was added at indicated concentrations, and incubated for 24 h prior to enumeration of viable bacteria. (G) Time course of survival of wild-type, *ΔcopA*, and *ΔcopA/*p*copA* strains pre-treated with copper (+Cu) or controls. Median ± interquartile range of CFU/ml from 3 independent experiments is presented. Dashed line, limit of detection (1000 or 10 CFU/ml). *P<0.*05*, **P<0.*01*, ***P<0.*001*, ****P<0.*0001*, Mann-Whitney test.

Wild-type and copper efflux-defective mutant strains were cultured in LB with copper to early-logarithmic phase. Menadione, an established intracellular superoxide generator in *E*. *coli* [25], was added and bacterial survival was determined after 24 h. Contrary to our prediction, we observed higher viable bacterial counts in cultures exposed to copper prior to addition of menadione, compared to controls (Fig 1). Copper-treated wild-type and *Δcus* mutant strains survived ~10-1000-fold higher than controls during menadione-induced superoxide stress (Fig 1A–1C). Copper rescued the survival of *ΔcopA* and *ΔcopAΔcus* mutants ~100,000-fold at 5 mM menadione (Fig 1B and 1D). Survival of wild-type and *Δcus* mutant strains was abolished to below the limit of detection (1000 CFU/ml) in medium containing 10 mM menadione. However, survival of *ΔcopA* and *ΔcopAΔcus* mutant strains at 10 mM menadione was ~100-fold higher in cultures pre-exposed to copper than controls (Fig 1B and 1D). To establish the specificity of the role of *copA* in this phenotype, we complemented *copA* expressed from its native promoter on a low-copy number plasmid (Table 1). Complementation rescued the survival of *ΔcopA* mutant in the presence of copper (S3 Fig). Copper-dependent protection from menadione killing in *ΔcopA* mutant did not change when transformed with empty vector (Fig 1E). Reintroduction of CopA in the *ΔcopA* mutant decreased the magnitude of protection observed at 5 mM menadione, and abolished protection at 2.5 and 7.5 mM menadione (Fig 1F). We also determined the temporal changes in UPEC survival after exposure to menadione (Fig 1G). Protective effect of pre-exposure to copper from menadione killing was not manifested at early time points (up to 6 hours), and becomes evident at 24 hours (Fig 1G). These results indicate that exposure to sublethal levels of copper promotes significant increase in bacterial survival during menadione-induced superoxide stress.

### Cellular copper and manganese content increases in UPEC exposed to copper

**Fig 2:**
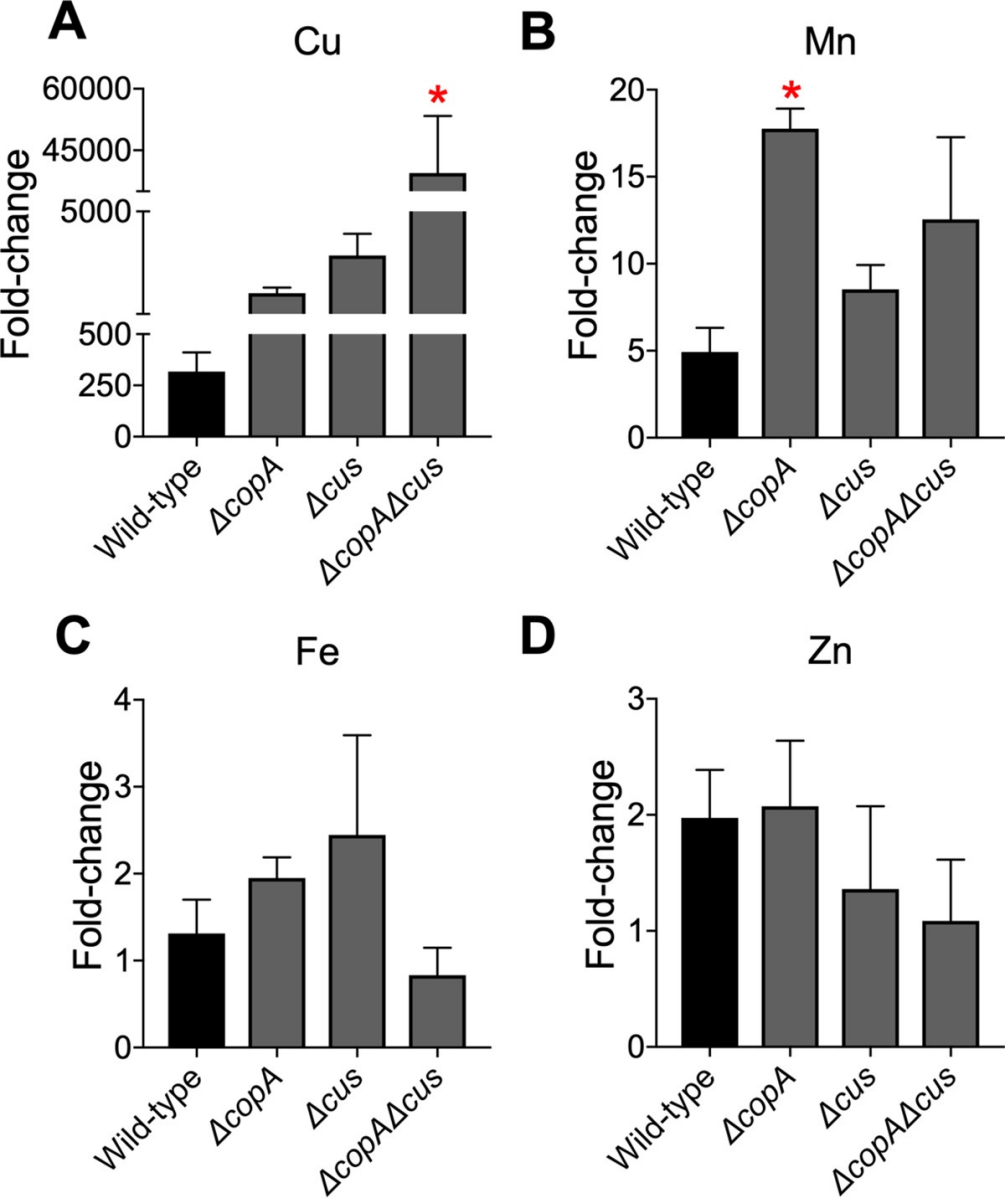
Increased copper content in UPEC cultured in copper-rich medium. Wild-type UPEC strain CFT073, and *ΔcopA*, *Δcus*, and *ΔcopAΔcus* mutants were grown in LB with or without copper to early logarithmic phase (OD_600_ = 0.3). Cell-associated copper (A), manganese (B), iron (C), and zinc (D) levels were determined by ICP-MS, and normalized to viable cell count. Mean + SEM of fold-change in metal content, relative to controls, from 3 independent experiments is depicted. *P<0.*05*, ANOVA with Dunnett’s multiple comparisons test.

We determined the effect of increased copper in the medium on the cellular levels of copper by ICP-MS. Since altered cellular copper content could affect the homeostasis of other metals, we also measured the levels of iron, manganese, and zinc. Wild-type, and isogenic mutants lacking copper efflux systems were cultured with and without copper. We noticed a remarkable increase in cell-associated copper content in all tested strains exposed to copper, compared to controls (Fig 2A). The *ΔcopAcus* double mutant had the highest fold-increase in cellular copper levels, followed by *Δcus*, *ΔcopA*, and the wild-type strain in descending order (Fig 2A). Differences observed in cellular copper content among the mutants (Fig 2A) are in agreement with the reported roles of CopA as an inner membrane transporter that exports copper from the cytoplasm to the periplasm, and CusCFBA as a transenvelope efflux system that removes excess copper from the periplasm [15,22]. Increased copper accumulation in the mutants, compared to parental wild-type strain, also served as a functional validation of these mutants (Fig 2A). Our results also reveal that exposure to high levels of copper results in a concurrent increase in the cell-associated manganese level in the *ΔcopA* mutant, compared to parental strain (Fig 2B). There was no discernable impact on intracellular iron and zinc accumulation in UPEC exposed to copper (Fig 2C and 2D).

### Copper increases the expression of copper efflux and manganese import genes

**Fig 3:**
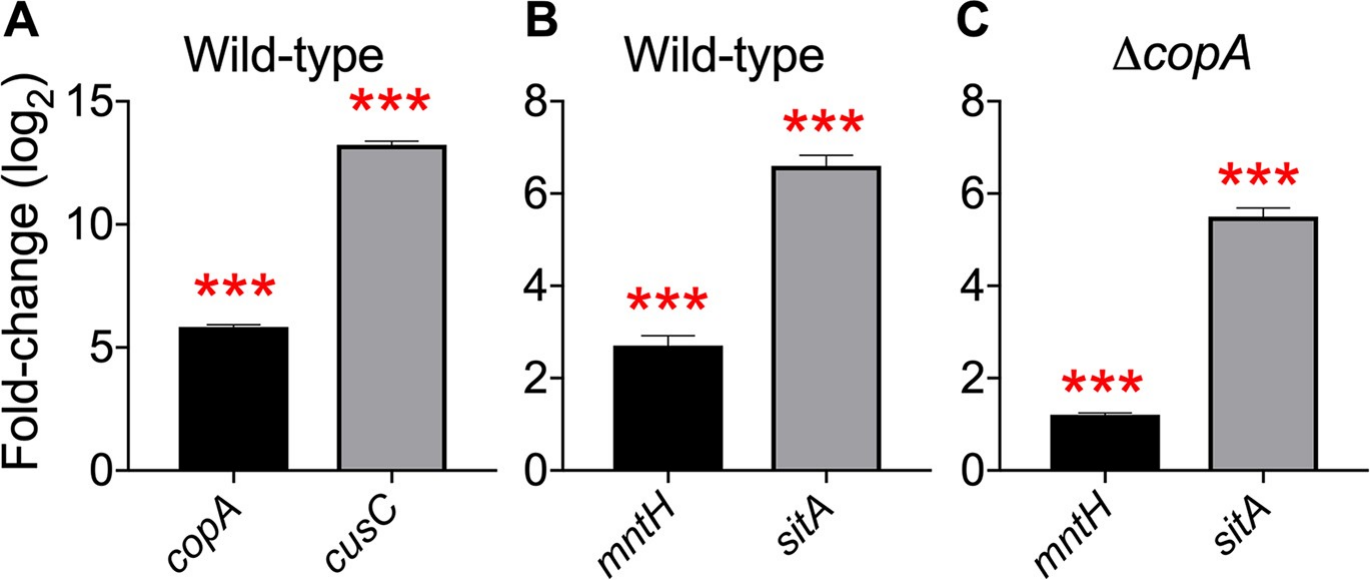
Copper induces the expression of copper efflux and manganese import genes. Wild-type UPEC strain CFT073 (A&B), or *ΔcopA* mutant (C) was cultured in LB with or without copper to early logarithmic phase (OD_600_ = 0.3). Real-time PCR was used to quantify *copA* (A), *cusC* (A), *mntH* (B&C), *sitA* (B&C) transcript levels. Relative expression, compared to no copper control, was calculated after normalizing to *gapA* transcript. Bars correspond to mean + SEM from 3 independent experiments. ***P<0.*001*, *t-*test.

We determined the expression of *copA* and *cusC* genes in the wild-type strain cultured with and without copper by real-time PCR to evaluate whether increased copper observed by ICP-MS (Fig 2A) is found inside the cell. Expression of both *copA* and *cusC* genes were significantly upregulated in the presence of copper, indicating that excess copper is found in the cytoplasmic and periplasmic compartments (Fig 3A). Since copper accumulation was associated with an increase in manganese content, we also measured the levels of *mntH* and *sitA* transcripts. UPEC encodes *sitABCD* genes, in addition to *mntH*, to import manganese [26]. These genes are repressed by MntR and Fur in the presence of manganese and iron in *Salmonella enterica* [27]. OxyR activates the transcription of *mntH* in response to hydrogen peroxide stress in *E*. *coli* and *S*. *enterica* [28,29]. We observed a significant increase in the abundance of *mntH* and *sitA* transcripts in the wild-type and *ΔcopA* strain exposed to copper, compared to controls (Fig 3B and 3C).

### Copper upregulates the expression of *sod* genes

**Fig 4:**
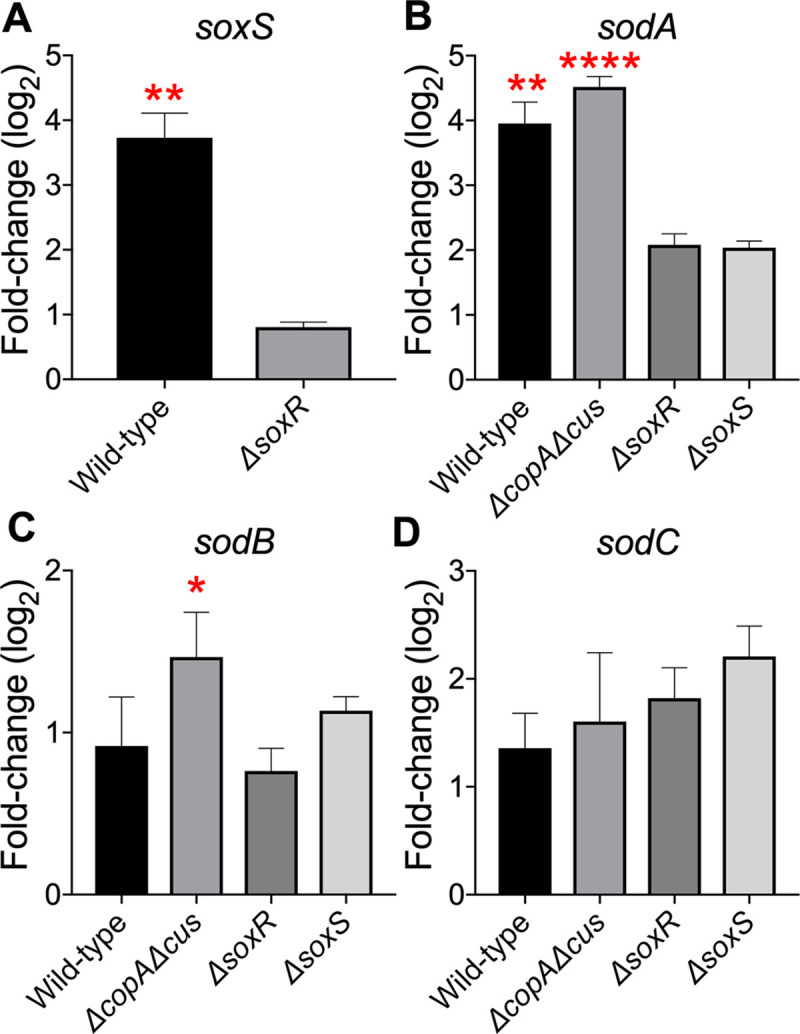
Expression of *sod* genes during sublethal copper stress. Wild-type UPEC strain CFT073, *ΔcopAΔcus*, *ΔsoxR*, and *ΔsoxS* mutants were grown in LB with or without copper. Real-time PCR was used to quantify *soxS* (A), *sodA* (B), *sodB* (C), *sodC* (D) transcript levels. Relative expression, compared to no copper control, was calculated after normalizing to *gapA* transcript. Bars correspond to mean + SEM from 3 independent experiments. *P<0.*05*, **P<0.*01* and ****P<0.*0001*, ANOVA with Dunnett’s multiple comparisons test.

*E*. *coli* encoded *sodA*, *sodB*, and *sodC* produce manganese-, iron-, and copper-zinc SODs, respectively. Since copper protects UPEC from killing by menadione, we tested whether copper affects the expression of *sodA*, *sodB*, *sodC* and *soxS* genes. SoxR is a sensor that promotes transcription of *soxS* in the presence of redox-cycling molecules that generate superoxide [30,31]. SoxS is a transcription factor that then activates the expression of multiple genes in the SoxRS regulon, including *sodA* [32]. Bacterial strains grown in LB to mid-logarithmic phase were exposed to sublethal levels of copper, and the expression of *sodA*, *sodB*, *sodC*, and *soxS* genes was quantified by real-time PCR. First, we validated this assay using menadione. The expression of *sodA* transcript in wild-type strain was 17 ± 3-fold higher in menadione-treated cells, as expected, compared to controls (S4 Fig). Induction of *sodA* expression was diminished in *ΔsoxR* and *ΔsoxS* mutants exposed to menadione (S4 Fig). Copper induced a 13 ± 4-fold increase in *soxS* transcript levels in the wild-type strain in a *soxR-*dependent manner (Fig 4A). The expression of *sodA* in wild-type and *ΔcopAΔcus* strains was 16 ± 4- and 23 ± 3-fold higher than the uninduced control, respectively (Fig 4B). Copper also activated *sodA* expression by ~4-fold in *ΔsoxR* and *ΔsoxS* strains, but this difference was not statistically significant (Fig 4B). There was a modest copper-dependent increase in *sodB* mRNA level in the *ΔcopAcus* double mutant (Fig 4C). Our results demonstrate that copper induces a robust increase in the abundance of *soxS* and *sodA* transcripts in UPEC.

### Activity of superoxide dismutases, SodA and SodC, is increased by copper

**Fig 5:**
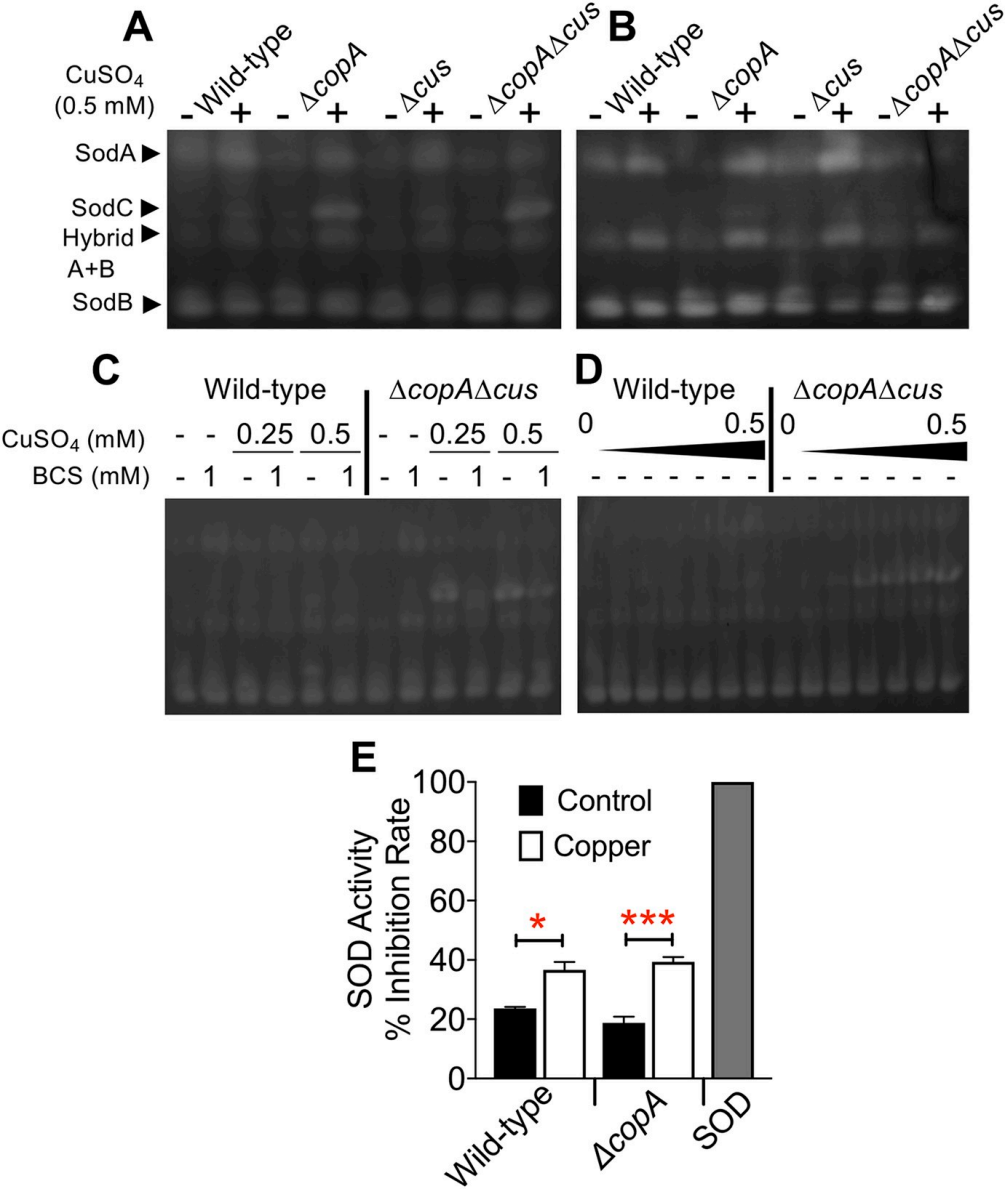
Copper induces the activity of SodA and SodC superoxide dismutases. Superoxide dismutase (SOD) activity in lysates of wild-type UPEC strain CFT073, *ΔcopA*, *Δcus* and *ΔcopAΔcus* mutants on non-denaturing polyacrylamide gels. (A) SOD activity in wild-type and copper efflux-defective mutants cultured with or without copper. (B) Sodium cyanide inhibits the activity of SodC. (C) Bathocuproine sulfonate (BCS), a copper chelator, inhibits copper-induced increase in SodC and SodA activity. (D) SOD activity at various concentrations of copper. (E) SOD activity of wild-type and *ΔcopA* mutant strains with and without pre-exposure to copper was determined using a nitroblue tetrazolium reduction assay. Bovine Cu-Zn SOD (SOD) was used as a positive control. Experiments were repeated at least 3 times. Representative images (A-D) and mean + SEM (E) are depicted. *P<0.*05*, and ***P<0.*0001*, ANOVA with Bonferroni’s multiple comparisons test.

We tested whether copper-dependent increase in superoxide resistance was attributable to increased SOD activity. Zymograms were performed with lysates of wild-type and copper efflux-defective mutants exposed to copper. Cyanide and bathocuproine sulfonate (BCS) were used as an inhibitor of SodC activity, and a specific chelator of copper, respectively [33–35]. The migration pattern of SODs on non-denaturing gels in our study (Fig 5) was consistent with the description of *E*. *coli* in-gel SOD activity from a previous report [34]. Activity of SodA and SodC, but not SodB, was enhanced in all strains exposed to copper (Fig 5A). This copper-dependent increase in SodA activity was consistent with our observation on the significant increase in *sodA* transcript levels (Fig 4B). Lack of increase in SodB activity (Fig 5A) during exposure to copper was compatible with a minimal change in transcript level (Fig 4C). A drastic increase in SodC activity was evident upon exposure to copper (Fig 5A), although copper did not affect the level of *sodC* transcript (Fig 4D). Notably, induction of SodC activity was higher in *ΔcopA* and *ΔcopAΔcus* mutants compared to wild-type and *Δcus* mutant strains (Fig 5A). The identity of SodC was confirmed by inhibition of its activity with cyanide (Fig 5B). Addition of BCS abrogated the ability of copper to induce both SodA and SodC in UPEC (Fig 5C). While a dose-dependent increase in the activity of SodC in response to copper was evident, a dose-response was less obvious for SodA (Fig 5D). Total SOD activity in wild-type and *ΔcopA* mutant strains was quantified by inhibition of reduction of NBT by superoxide generated from xanthine-xanthine oxidase reaction. Exposure to copper resulted in a significant increase in SOD activity in wild-type and *ΔcopA* mutant strains (Fig 5E). Collectively, these experiments indicate that exposure to copper is a strong inducer of the activity of SodA and SodC, but not SodB, in UPEC.

### SodA is required for copper-dependent resistance to killing by superoxide

**Fig 6:**
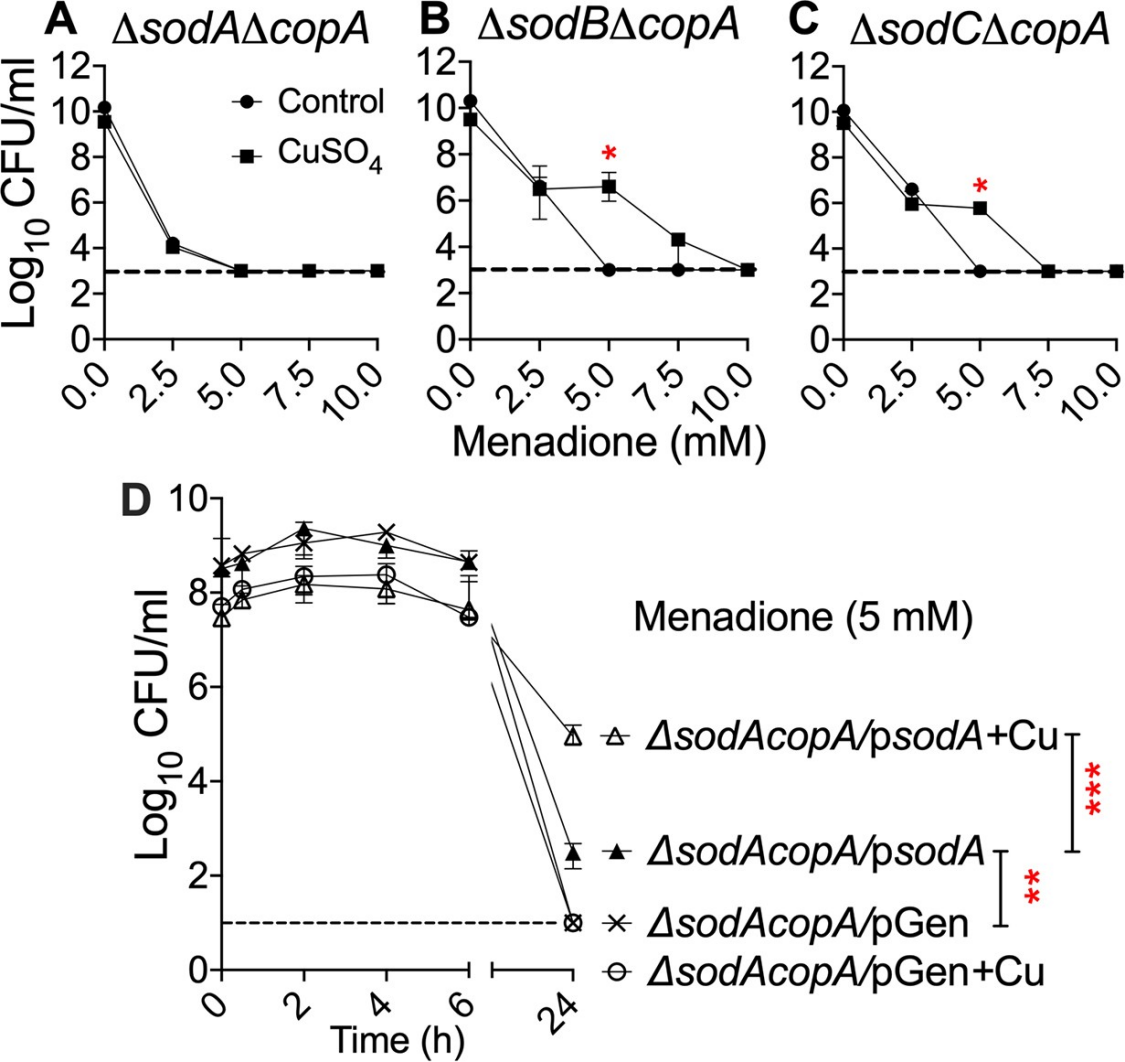
Copper-induced protection from killing by superoxide is dependent on *sodA*. Double mutants *ΔsodAΔcopA* (A), *ΔsodBΔcopA* (B) and *ΔsodCΔcopA* (C) were grown in LB (circles) and LB with copper (squares) to early logarithmic phase. Menadione was added at indicated concentrations, and viable count was determined after 24 hours. (D) Time course of survival of *ΔsodAcopA* mutant with empty vector (pGen) or complemented with *sodA (*p*sodA)*. Strains were pre-treated with copper (+Cu) or control prior to addition of menadione (5 mM). Median ± interquartile range of CFU/ml from 3 independent experiments is depicted. Dotted line, limit of detection (1000 or 10 CFU/ml). *P<0.*05*, **P<0.*01*, and ***P<0.*001*, Mann-Whitney test.

UPEC mutants lacking individual SODs were validated by testing their sensitivity to menadione-induced superoxide stress under aerobic and anaerobic conditions. Under aerobic condition, *sodA* is indispensable for protection from killing by superoxide (S5A Fig). As anticipated, this effect was lost under anaerobiosis (S5B Fig). Loss of *sodB* and *sodC* did not alter the sensitivity of UPEC to superoxide, and was comparable to wild-type strain (Fig S5A and S5B). Double mutants *ΔsodAΔcopA*, *ΔsodBΔcopA* and *ΔsodCΔcopA* were constructed to delineate the impact of individual SOD on copper-dependent protection from killing by superoxide. Menadione resistance of *ΔsodA* and *ΔsodAΔcopA* mutants was rescued upon reintroduction of *sodA* on a low-copy number plasmid (S6 Fig). We determined the superoxide sensitivity of these double mutants exposed to copper, and untreated controls. Protective effect of copper from superoxide killing (Fig 1B), was completely abrogated in *ΔsodAΔcopA* mutant (Fig 6A). Lowering the limit of detection from 1000 cfu/ml (Fig 6A) to 10 cfu/ml (S7 Fig) also confirmed the absence of a detectable effect of copper on promoting resistance to superoxide in the *ΔsodAΔcopA* mutant. Presence of SodA in *ΔsodBΔcopA* and *ΔsodCΔcopA* mutants conferred protection from 5 mM menadione (Fig 6B and 6C). However, protection at other concentrations of menadione observed in the *ΔcopA* mutant (Fig 1B) was not rescued in *ΔsodBΔcopA*, and *ΔsodCΔcopA* mutants (Fig 6B and 6C). Time course assay revealed that restoration of *sodA*, even in the absence of pre-exposure to copper, partially revives growth of the *ΔsodAΔcopA* mutant exposed to menadione (Fig 6D). Complemented mutant exhibited copper-dependent protection from killing by superoxide stress induced by menadione (Fig 6D). Copper dependent protection was evident at 24 hours, comparable to results depicted in Fig 1G. Our results reveal that SodA is the primary mediator of copper-dependent protection from killing by superoxide in *E*. *coli*. It also illustrates that the extent of this protection varies based on the intensity of superoxide stress.

### Role of SODs in UPEC fitness during UTI

**Fig 7:**
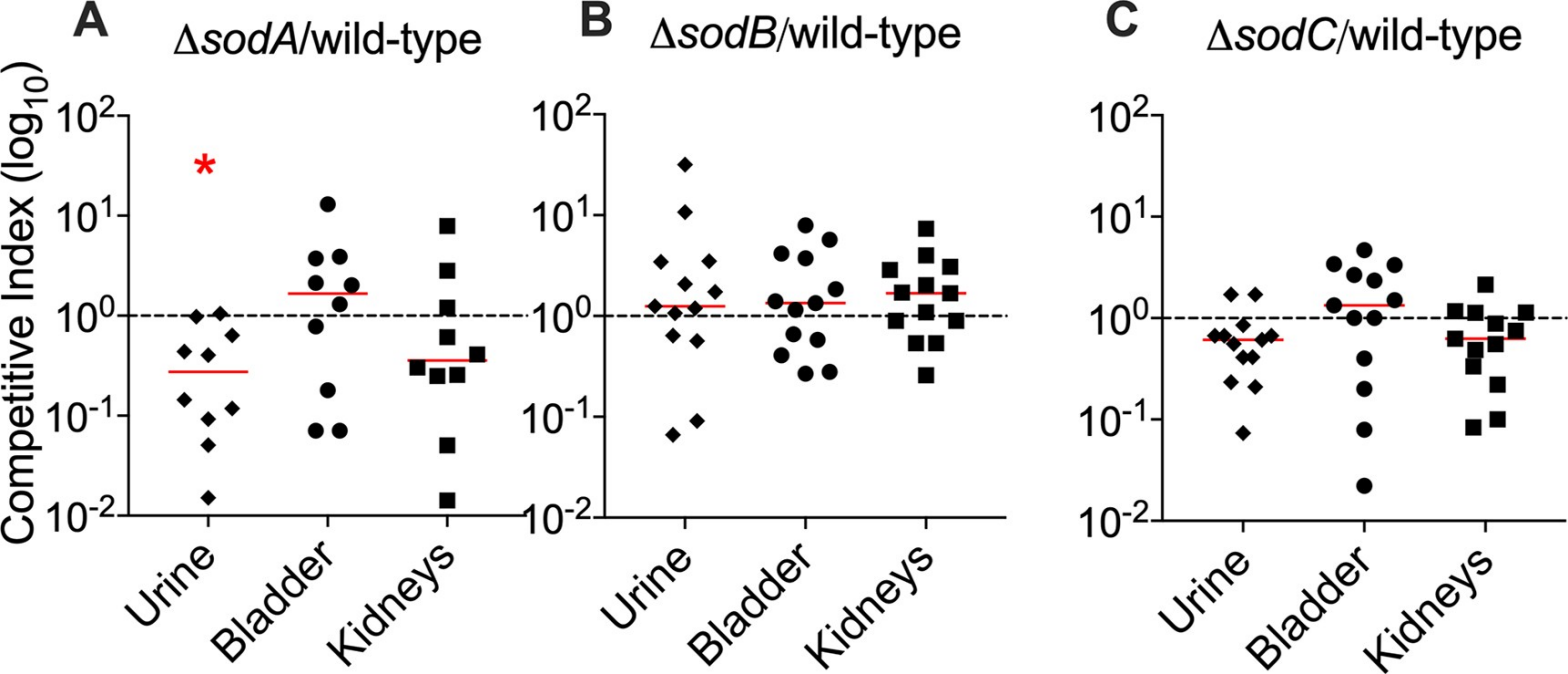
Role of *sod* genes in fitness of UPEC in the mouse model of UTI. Mice were inoculated with a mixture of wild-type strain and a mutant lacking *sodA* (A), *sodB* (B), or *sodC* (C) genes. Wild-type and mutant strains in the inoculum and tissue samples were enumerated. Competitive indices were calculated as the ratio of mutant to wild-type strain *in vivo*, and normalized to the ratio mutant to wild-type strain in the inoculum. Each symbol corresponds to results from a mouse, and bars indicate median. Dotted line, no loss of fitness in the mutant relative to parental wild-type strain (competitive index of 1). *P<0.05, Wilcoxon signed rank test.

Co-infection experiments were performed in the mouse model of UTI to determine the contribution of SODs to *in vivo* fitness of UPEC. Strains were cultured in LB, with no additional copper, prior to inoculation. Relative fitness of mutants lacking *sodA*, *sodB*, or *sodC* was compared to the parental strain. A significant decrease in fitness was observed for the *sodA* mutant in urine, where it was recovered ~5-fold lower than the wild-type strain (Fig 7A). There was no detectable change in fitness at other sites, or for *ΔsodB*, or *ΔsodC* mutants in the murine urinary tract (Fig 7A–7C).

### Copper primes *sodA*-dependent increase in intramacrophage survival of UPEC

**Fig 8:**
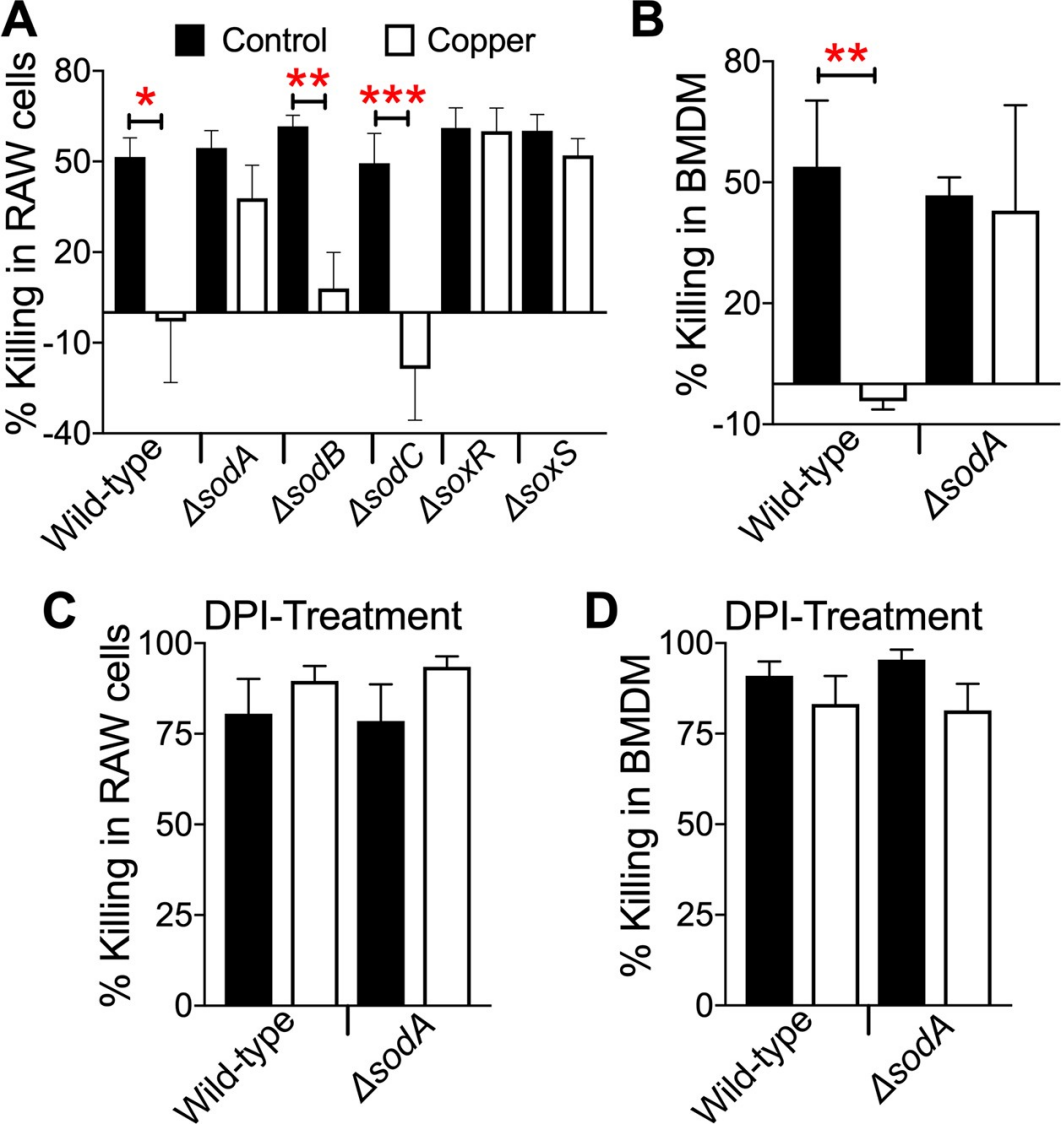
UPEC *sodA* mediates copper-dependent protection from killing within macrophages. (A) RAW264.7 murine macrophages were infected with copper-exposed or control wild-type strain CFT073, and *ΔsodA*, *ΔsodB*, *ΔsodC*, *ΔsoxR*, *or ΔsoxS* mutants. Extracellular bacteria were killed with gentamicin. *E*. *coli* internalization, and survival within macrophages was determined by viable counts. Bacterial counts were normalized to protein content, and percent killing was calculated. (B) Percent killing of copper-exposed or control wild-type, and *ΔsodA* mutant strains in murine bone marrow-derived macrophages (BMDM). Percent bacterial killing in RAW cells (C) and BMDMs (D) treated with diphenyleneiodonium (DPI, 20 μM), an inhibitor of NADPH oxidase. Bars depict mean + SEM from 3–6 independent experiments, conducted in triplicate. *P<0.*05*, **P<0.*01*, ***P<0.*001*, ANOVA with Bonferroni’s multiple comparisons test.

We sought to determine the impact of pre-exposure to copper on bacterial survival within macrophages, since both copper and superoxide are antibacterial agents deployed inside the phagolysosomes [18]. Gamma interferon (IFN-γ)-stimulated RAW264.7 murine macrophages and bone marrow-derived macrophages (BMDMs) were infected with wild-type, *ΔsodA*, *ΔsodB*, *ΔsodC*, *ΔsoxR*, or *ΔsoxS* mutant strains cultured in LB with or without copper. Gentamicin protection assays were performed to determine intramacrophage killing of these strains. Mutants exhibited comparable level of intramacrophage killing (~55%) to the wild-type strain, when cultured in medium without added copper (Fig 8A). In contrast, killing within IFN-γ-stimulated macrophages was eliminated for copper-exposed wild-type strain (Fig 8A). This copper-dependent protective effect was lost in the *ΔsodA*, *ΔsoxR*, and *ΔsoxS* mutants as there was no statistically significant difference in the percent killing between copper-treated, and control bacterial cells (Fig 8A). Copper-dependent decrease in intramacrophage killing was observed in *ΔsodB* and *ΔsodC* mutants, and was comparable to that of the wild-type strain (Fig 8A). To expand the *in vivo* relevance, this bacterial killing assay was repeated in BMDMs. Gentamicin protection assays in BMDMs reveal that wild-type UPEC pre-exposed to copper is protected from killing (Fig 8B). This protective effect was lost in the *ΔsodA* mutant (Fig 8B), comparable to the findings in RAW cells (Fig 8A). We tested whether copper-induced protection for UPEC from killing by macrophages was dependent on ROS generation during oxidative burst. Diphenyleneiodonium (DPI) is an inhibitor of NADPH oxidase that has been used to abrogate superoxide generation in macrophages [36], including in UPEC-macrophage interaction studies [37]. DPI treatment abrogated copper-dependent protection in the wild-type strain in RAW264.7 cells and BMDMs (Fig 8C and 8D). Our results revealed an overall increase in bacterial killing during DPI treatment (Fig 8C and 8D), consistent with previous reports including in UPEC [37]. Taken together, our results indicate that copper primes UPEC for protection within macrophages, and this protective effect depends on the presence of *sodA*.

## Discussion

**Fig 9:**
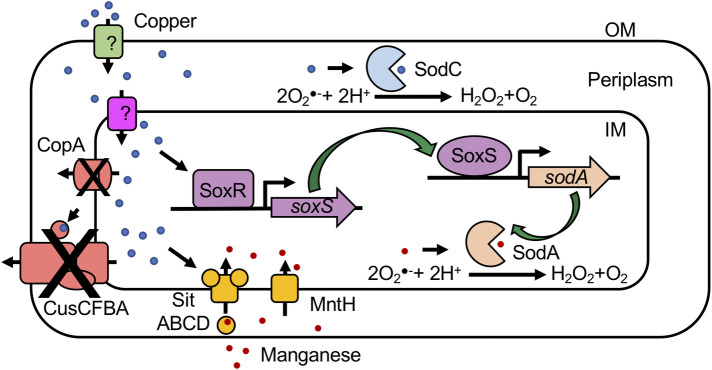
Working model of copper-dependent protection of UPEC from superoxide. High levels of copper in the environment results in increased cellular copper content. Copper stress increases the expression of copper efflux and manganese import genes. Accumulation of copper and manganese is exaggerated in copper-efflux defective mutants. Activation of SoxR culminates in increased SodA activity via induction of SoxS-dependent transcription of *sodA*. Copper stress also enhances the activity of SodC. Collectively, these copper-dependent changes impart protection from killing by superoxide. IM, inner membrane; and OM, outer membrane.

Copper and superoxide are both toxic to *E*. *coli* independently. We anticipated a double-hit scenario where superoxide would be additively or synergistically deleterious to copper-replete UPEC. In contrast, our results demonstrate that pre-exposure to sublethal concentration of copper acts via SodA and SodC to promote protection of *E*. *coli* from killing by superoxide (Fig 9). In the context of host-pathogen interaction, pre-exposure to copper mitigates the killing of UPEC within murine macrophages. Furthermore, higher protection from superoxide was observed in copper efflux-defective *ΔcopA* and *ΔcopAΔcus* mutants that have increased intracellular copper content, than parental UPEC strain. Copper stress also increases the expression of manganese import genes, and elevates the levels of cell-associated manganese. Collectively, our results suggest a mechanistic model in which copper triggers the activation of SodA and SodC through independent mechanisms to protect UPEC from killing by superoxide, a critical antibacterial effector generated during oxidative burst in macrophages (Fig 9). These significant findings shed new light on the effects of copper, and adaptation to oxidative stress by establishing a direct role for copper in protection against superoxide stress in *E*. *coli*. A major ramification of our findings is that bacterial residents of niches with higher copper content, such as the gut, are primed for resistance against killing by superoxide within phagocytes.

CopA, CueO and CusCFBA are critical effectors of copper detoxification in *E*. *coli* [15,22,38]. CueO is a copper-containing periplasmic multicopper oxidase that oxidizes more toxic cuprous ions to less toxic cupric ions [38]. A UPEC mutant lacking CueO is more virulent in the mouse model of UTI due to its increased ability to acquire iron [39]. We used *ΔcopA*, *Δcus* and *ΔcopAΔcus* mutants, but not a *ΔcueO* mutant, in this study. This was based on our interest in the impact of augmented cytosolic copper content on superoxide stress, and also to avoid increased iron content as a confounding factor in our results. Differences in copper content observed in copper efflux-defective mutant strains (Fig 2A) is consistent with their reported function of CopA removing excess copper from the cytoplasm, and CusCFBA removing excess copper from the periplasm [15,22].

The SoxRS regulatory system orchestrates the expression of genes involved in responding to, and survival under superoxide stress in *E*. *coli* [40]. In a previous study, we had noticed increased transcription of *soxR*, and *soxS* in three out of five UPEC isolates during UTI in patients, where it is exposed to copper [7]. Evidence generated in this study demonstrates that copper activates SoxR*-*mediated transcription of *soxS* thereby increasing *sodA* transcript levels, enhancing SodA activity, and a SodA-dependent protection from killing by superoxide in UPEC (Fig 9). Copper could activate SoxR directly, independent of superoxide, in a mechanism analogous to that described for redox-cycling drugs in *E*. *coli* [31]. Our results are congruent with, and extend previous findings on copper and superoxide stress in *E*. *coli* [14]. Microarray-based transcriptome studies have revealed that copper triggers expression of genes in the SoxRS regulon in *E*. *coli* [12,13]. Interestingly, the magnitude of induction of *sodA* transcription was decreased, but not eliminated, in *ΔsoxR* and *ΔsoxS* mutants exposed to copper (Fig 4B), and also menadione (S4 Fig). MarA, a transcriptional regulator of resistance to antibiotics and other stressors, has been implicated in the transcriptional regulation of *sodA* in *E*. *coli* [41]. Taken in light of our results, SoxRS-independent transcription of *sodA* could be driven by MarA in the presence of copper emerges as a hypothesis that remains to be tested.

SodC is a periplasmic copper-zinc SOD found in Gram-negative bacteria, including *E*. *coli* and *S*. *enterica* [42,43]. Highly-pathogenic isolates of enterohemorrhagic *E*. *coli* but not UPEC, and *S*. *enterica* carry additional copies of *sodC* [44,45], underscoring the association of *sodC* with virulence. Expression of *sodC* in *E*. *coli* is regulated by RpoS, the sigma factor governing transcriptional response during stationary phase [43,46]. UPEC isolates express RpoS in both logarithmic and stationary phases, unlike the K-12 strains that express RpoS only in stationary phase [47]. This supports our observation of induction of SodC activity in UPEC in logarithmic phase. We did not find statistically significant changes in *sodC* transcript level in wild-type, copper efflux-defective, *soxR*, and *soxS* mutant strains exposed to copper (Fig 4D). Gort *et al*. found that chelation of copper leads to decreased transcription of *sodC* [43]. A UPEC mutant lacking *rpoS* is attenuated in a host ROS-dependent manner in the mouse model of UTI [48]. Our results do not indicate a role for *sodC* in the fitness of UPEC during UTI (Fig 7C). Taken together, decreased virulence of the UPEC mutant lacking RpoS is more likely due to loss of function of non-*sodC* members of its regulon. Additional studies are necessary to determine the extent to which copper impacts expression of genes in the RpoS regulon, and its impact on the pathogenesis of UPEC-induced UTI.

Current understanding is that unfolded apo-SodC is secreted into the periplasm by general secretory pathway to be metallated in that milieu in *E*. *coli* [49]. The presence and identity of periplasmic chaperone(s) that deliver copper to SodC in *E*. *coli* is not known. CueP is a periplasmic copper chaperone that trafficks copper exported by CopA and GolT, another inner membrane copper efflux pump found in *Salmonella* but not in *Escherichia*, ATPases to metallate SodCII in *S*. *enterica* serovar Typhimurium [50]. In *E*. *coli*, CusF is a periplasmic copper chaperone that aids copper efflux via the CusCBA system [51]. Whether CusF plays additional roles in delivering copper to periplasmic cuproproteins remains to be determined in *E*. *coli*. Since SodC activity is enhanced in mutants lacking both CopA and CusCFBA exposed to copper (Fig 5A), it is tempting to speculate that copper delivery via CopA and/or CusF is not involved in metallation of SodC in *E*. *coli*. It must be noted that our experiments were not designed to address the role of CopA and CusF in copper trafficking to SodC. Since SOD activity assays were performed after induction with high levels of copper (up to 0.5 mM, Fig 5A–5D), the role of CopA and/or CusF in metallation of SodC could have been masked. Apparent disconnect between lack of increase in *sodC* transcript level (Fig 4D), and a robust increase in SodC activity (Fig 5) suggests that post-transcriptional regulatory mechanisms, such as metallation, could serve as important modulators of SodC activity under copper-replete conditions.

Here, we evaluated the impact of bacterial exposure to copper prior to encountering macrophages, and found that copper enhances UPEC survival within murine macrophages in a *sodA-*dependent manner (Fig 8). SodA is required for successful survival of *S*. *enterica* serovar Typhimurium within macrophages, and to resist ROS generated by murine neutrophils [52,53]. Our study illustrates that bacteria exposed to copper prior to phagocytosis are better adapted to resist killing by macrophages *via* induction of SodA activity in *E*. *coli*. Importantly, copper-dependent protection mediated by SodA in killing by macrophages is abrogated upon inhibition of ROS generation (Fig 8C and 8D). The role of cytoplasmic SodA in protection from superoxide-dependent killing in macrophages could be puzzling because extracellular superoxide anion does not normally cross lipid bilayers to enter the cytoplasm. However, superoxide readily penetrates lipid bilayers when pH is <7, found in *E*. *coli*-containing phagolysosomes, because it is not charged, and is protonated [54]. This finding provides a framework for understanding the role of cytoplasmic SodA in protection from superoxide-mediated killing by phagocytes in our study. Studies on *E*. *coli*, *S*. *enterica*, and *Mycobacterium avium* have, however, revealed copper as an antibacterial effector within macrophages harboring phagocytosed bacteria [11,17–19,55]. Macrophages pre-treated with copper kill a UPEC mutant lacking yersiniabactin at a higher level than wild-type UTI89 strain [37]. Yersiniabactin is a siderophore, and a cuprophore produced by UPEC isolates and strains, including prototypical strains UTI89 and 536 [8,56–58]. UPEC lacking yersiniabactin-biosynthetic potential, including another prototypical UPEC strain CFT073 used in this study, are also virulent in the mouse model of ascending UTI [59]. Additionally, copper-yersiniabactin complexes display a novel SOD-like activity that mitigates killing of UPEC by ROS [37]. In summary, copper activates SOD-dependent and yersiniabactin-dependent superoxide detoxification systems in UPEC.

Our study offers mechanistic insights into the role of copper in protection from superoxide stress in *E*. *coli*. Hydrogen peroxide, another inducer of oxidative stress, is produced during dismutation of superoxide (Fig 9). Hydrogen peroxide is highly lethal and needs to be detoxified. Increased levels of hydrogen peroxide trigger the expression of OxyR-regulated genes [60,61]. Macomber and Imlay have demonstrated that copper plays a key role in protection against DNA damage caused by hydrogen peroxide in *E*. *coli* [62]. Copper induces the activity of catalases KatE (regulated by RpoS) and KatG (regulated by OxyR) in *E*. *coli* [62]. Taken together, copper emerges as an activator of protective responses against both hydrogen peroxide and superoxide, important antibacterial effectors of phagocytes, in *E*. *coli*. While our findings offer compelling evidence for the role of copper in protection from superoxide toxicity in *E*. *coli*, it also generates several important questions. Whether copper activates SoxR directly or indirectly via superoxide is an important question that requires investigation. A logical extension of this work is to explore whether this response is conserved in other bacterial pathogens, particularly in *Salmonella* and other members of *Enterobacteriaceae*. Mechanism of copper delivery to metallate SodC in the periplasm of *E*. *coli* is also a key question that needs to be addressed. Additional questions pertaining to environmental factors such as pH, presence of reductants, and oxygenation on copper-dependent protection from killing by superoxide must also be investigated to elucidate the scope and impact of changes induced by copper in bacteria.

## Materials and methods

### Bacterial strains and mutant construction

Clinical UPEC strain CFT073 was isolated from the urine and blood of patient [63]. Targeted mutations to disrupt genes in involved in copper efflux (*copA* and *cusSRCFBA*) and superoxide dismutation (*sodA*, *sodB*, and *sodC*) were introduced into UPEC strain CFT073 by lambda red recombineering [64]). Successful introduction of mutations was verified by PCR with primers that bind to kanamycin or chloramphenicol resistance cassette, and gene-of-interest. Strains and oligonucleotide primers used in this study are listed in Table 1 and S1 Table, respectively.

### Complementation

Genes including upstream regions containing CueR binding site for *copA* and SoxS binding site for *sodA* were synthesized (Genscript), and cloned into pGEN-MCS [65] by ligation at BamHI and SalI sites. Constructs were verified by sequencing, and electroporated into mutant strains. Plasmids used in this study are listed in Table 1.

### Culture conditions and reagents

Strains were cultured in LB broth or agar (tryptone 10 g/l; yeast extract 5 g/l; NaCl 5 g/l; and agar 15 g/l). Cultures were incubated at 37°C, and aerated by shaking at 200 RPM, unless noted otherwise. Early- and mid-logarithmic phases correspond to an OD_600_ of 0.3 and 0.5, respectively. Chemicals were purchased from Sigma, and exceptions are noted throughout this section.

### Menadione killing assay (broth)

Bacterial cultures were grown in LB with or without 2 mM CuSO_4_ to early-logarithmic phase (OD_600_ = 0.3). Menadione (0, 2.5, 5, 7.5 or 10 mM) was added to these cultures, and incubated for 24 hours. Viable bacterial counts were enumerated on LB agar.

### Menadione killing assay (agar)

Bacterial cells in mid-logarithmic phase (OD_600_ = 0.5) were serially diluted, and deposited on LB agar with menadione (0, 0.5, 1, or 1.5 mM). Bacterial counts were determined after overnight incubation under aerobic or anaerobic conditions (GasPak, BD). Percent survival was calculated as the ratio of number of bacteria in menadione treatment to control LB plate, and multiplied by 100.

### Copper killing assay (agar)

This assay was carried out essentially as described above for menadione, but using CuSO_4_ (0, 0.5, 1, 2, 2.5, 3 or 5 mM), and cells in mid-logarithmic (OD_600_ = 0.5) and stationary phases.

### ICP-MS

Bacterial cultures were grown in LB (10 ml) with or without 2 mM CuSO_4_ to early logarithmic phase (OD_600_ = 0.3). Bacterial cells were harvested by centrifugation, and washed with HEPES (10 mM, pH 7.4) containing 0.5 mM EDTA. Washing was repeated with HEPES (10 mM, pH 7.4). Cell pellets were digested with 200 μl of nitric acid (trace element-grade) at 100°C for an hour. Copper, manganese, iron and zinc levels were determined by ICP-MS (8800 Triple Quadrupole, Agilent Technologies) by an operator blinded to sample identity. Analysis was conducted in a single quadrupole mode using helium in the collision/reaction cell to minimize spectral interference. Concentration of trace elements was normalized to nanogram per million CFU of *E*. *coli*, and fold-change in metal content was calculated relative to untreated controls.

Colon contents were collected from mice used for harvesting bone marrow. Colons were excised, their contents were carefully extruded. Samples were digested in nitric acid at 100°C for two hours, diluted in trace elements-grade water prior to determining copper levels by ICP-MS, as described above, and normalized to μg/g of wet weight of colon content.

### Quantitative PCR

Bacterial cultures were grown to mid-logarithmic phase (OD_600_ = 0.5), and 0.5 mM menadione or CuSO_4_ was added. 2 mM CuSO_4_ was used to determine expression of copper efflux and manganese import genes (Fig 3). RNAprotect (Qiagen) was added after 20 minutes to stabilize the transcripts, and cells were harvested by centrifugation. RNA was extracted with RNeasy mini kit (Qiagen), and treated with DNase (Turbo DNA-free, Ambion) to eliminate contaminating DNA. cDNA was synthesized with Superscript III reverse transcriptase (Invitrogen). SYBR green-based qPCR (Maxima master mix, Thermo Scientific) was performed with oligonucleotide primers, listed in S1 Table, in a CFX Real-Time system (Bio-Rad Laboratories). Transcript levels were normalized to *gapA* mRNA. Relative expression was determined using untreated controls of each strain as calibrator.

### SOD zymograms

Cells in mid-logarithmic phase (OD_600_ = 0.5) were induced with indicated concentration of CuSO_4_ for 30 minutes. Bacterial cells were harvested by centrifugation, resuspended in a Tris-based buffer (10 mM Tris-HCl pH 8.0, 0.6 M sucrose, and 0.1 M EDTA), and lysed by sonication. Total protein (100 μg) from clarified supernatants was separated on non-denaturing, 10% polyacrylamide gels by electrophoresis. SOD activities were determined by staining with nitroblue tetrazolium (NBT, 2.43 mM), TEMED (28 mM), riboflavin-5’-phosphate (28 mM) in phosphate buffer (pH 7.8) for 20 minutes at room temperature [35]. The sensitivity of SODs to sodium cyanide (NaCN) was determined by staining the gels in the presence of 10 mM NaCN [35]. The SOD activity bands were developed under fluorescent light, and imaged in a Gel Doc system (Bio-Rad Laboratories).

### SOD activity

Total SOD activity was determined as described previously [66]. Bacterial strains in mid-logarithmic phase (OD_600_ = 0.5) were induced with 0.5 mM CuSO_4_ or water for 30 minutes. Cells were harvested, lysed by sonication, clarified by centrifugation and protein content was measured (BCA assay). 100 μg of extract of wild-type strain and *ΔcopA* mutant was added to the assay mixture composed of 100 mM potassium phosphate buffer (pH 8.0), 0.1 mM xanthine, 0.1 mM EDTA, 0.025 mM NBT and 0.5% BSA. The reaction was initiated by addition of 60 mU/ml of xanthine oxidase. Reduction of NBT was monitored at 560 nm for 1 hour, and inhibition rate of NBT reduction was calculated. Recombinant bovine Cu-Zn SOD was used as a positive control at 10 ng/ml.

### Bacterial killing in RAW264.7 mouse macrophages

Gentamicin protection assays were conducted as described previously [67]. Briefly, immortalized murine macrophages (RAW 264.7, ATCC TIB-71) were maintained in Gibco RPMI 1640 medium (Invitrogen) containing fetal bovine serum (10% v/v, Invitrogen), and penicillin, streptomycin and glutamine (1% v/v, Invitrogen). RAW264.7 cells were seeded at 2.5x10^5^ cells/well in 24-well plates, and stimulated with murine IFN-γ (100 ng/ml, Millipore) for 24 hours. UPEC strains were grown to mid-logarithmic phase (OD_600_ = 0.5) in LB with or without 2 mM CuSO_4_, washed and resuspended in PBS to remove extracellular copper. Diphenyleneiodonium (DPI, 20 μM) was added during infection, and maintained until endpoints. Bacteria were added to RAW264.7 cells at a multiplicity of infection of three, and briefly centrifuged to synchronize uptake. Fresh medium with gentamicin (100 μg/ml) was added 30 minutes post-infection to kill extracellular bacteria. Gentamicin was removed after 15 minutes (T0), and 75 minutes (T1). Cells were lysed with 0.1% triton X-100 to determine intracellular bacterial load. Bacterial counts were normalized to protein content/well. Percent killing was calculated from the number of *E*. *coli* killed within RAW264.7 cells at T1, adjusted for the number of internalized bacteria at T0.

### Ethics statement

All procedures involving the use of mice were approved by the Institutional Animal Care and Use Committee at Texas A&M University (protocol #2018–0362). Mice were provided access to food and water ad libitum. Tribromoethanol (250 mg/kg) was used for anesthesia, and isoflurane was used for euthanasia.

### Bacterial killing in mouse BMDMs

Adult female C57BL/6 mice (N = 5, 4–7 weeks-old, Jackson Laboratories) were euthanized, and bone marrow was collected from femurs and tibia. BMDMs were generated as described previously [68]. Briefly, erythrocytes in bone marrow cell suspension were lysed, and remaining cells were cultured in the presence of recombinant murine M-CSF (Invitrogen) at 25 ng/ml for seven days to differentiate BMDMs. Fresh media was added on day 3. Bacterial killing assays with BMDMs were conducted essentially as described above for RAW264.7 cells, with a multiplicity of infection of two.

### Mouse model of UTI

Adult female CBA/J mice (4–6 weeks-old, Jackson Laboratories) were used for co-infection with wild-type and mutant strains, as described previously [7]. Briefly, 10^8^ CFU of 1:1 mixture containing wild-type UPEC strain CFT073, and *ΔsodA*, *ΔsodB*, or *ΔsodC* mutant were instilled in the urinary bladders. This experiment was repeated twice with 5–8 mice/group, independently. Urine samples were collected, and mice were euthanized at 48 hours post-inoculation. Organs were removed aseptically, homogenized, plated on LB agar with or without kanamycin, and incubated aerobically at 37°C to determine CFU/g or ml. Mutant strains grow on plain and kanamycin-containing agar, whereas wild-type strain grows only on plain agar. Wild-type and mutant bacteria surviving *in vivo* were enumerated to calculate competitive indices (CI). CI = urine or tissue (mutant cfu/ml or g / wild-type cfu/ml or g) / inoculum (mutant cfu/ml /wild-type cfu/ml). CI<1 indicates a fitness defect in the mutant, relative to parental strain.

### Statistical analysis

All experiments were repeated at least three times independently, with at least two technical replicates. Results were analyzed in Prism 7 (Graphpad) with *t*-test, Mann-Whitney test, ANOVA followed by Dunnett’s or Bonferroni’s multiple comparisons test, or Wilcoxon signed rank test. *P<0*.*05* was considered as a statistically significant difference. Error bars depicted in *Figures* correspond to SEM or interquartile range. Lack of error bars indicate that they are smaller than the symbol itself.

## Supporting information

S1 Table Oligonucleotide primers used in this study. (DOCX)

S1 Fig Copper sensitivity of UPEC strain CFT073, and its copper efflux-defective mutants. (TIF)

S2 Fig Copper levels in colonic content of mice. (TIF)

S3 Fig Genetic complementation rescues copper resistance in a *ΔcopA* mutant. (TIF)

S4 Fig Menadione induces the expression of *sodA*. (TIF)

S5 Fig *sodA* protects *E*. *coli* from killing by superoxide. (TIF)

S6 Fig Genetic complementation rescues menadione resistance in *ΔsodA* and *ΔsodAcopA* mutants. (TIF)

S7 Fig Copper does not rescue menadione resistance in a *ΔsodAcopA* double mutant. (TIF)
