# Supplementary material for: Copper primes adaptation of uropathogenic Escherichia coli to superoxide stress by activating superoxide dismutases
Source: PLoS Pathog. 2020 Aug 26;16(8):e1008856. doi: 10.1371/journal.ppat.1008856 (PMC7478841; doi:10.1371/journal.ppat.1008856)
Supplement: S1 Table — (DOCX) [file ppat.1008856.s001.docx]

**S1 Table. Oligonucleotide primers used in this study**

| **Primer ID^a^** | **Sequence** |
| --- | --- |
|  | **Purpose: Construction of mutants by Lambda Red recombination** |
| P216 *copA* F | 5'TTATTCCTTCGGTTTAAACCGCAGCAATCGGTTGGCGTTACTCACTACGGGTGTAGGCTGGAGCTGCTTC3' |
| P217 *copA* R | 5'ATGTCACAAACTATCGACCTGACCCTGGACGGCCTGTCCTGCGGTCACTGATGGGAATTAGCCATGGTCC3' |
| P307 *sodA* F | 5’AACCAACTGCTTACGCGGCATTAACAATCGGCCGCCCGACAACACTGGAGGTGTAGGCTGGAGCTGCTTC3' |
| P308 *sodA* R | 5'TTTAAGCTGCAATGCGGCGTAAACGCCTCATTGCAGCAGGCGGCAAATGAATGGGAATTAGCCATGGTCC3' |
| P309 *sodB* F | 5'CACAATAAGGCTATTGTACGTATGCAAATTAATAATAAAGGAGAGTAGCAGTGTAGGCTGGAGCTGCTTC3' |
| P310 *sodB* R | 5'TGGCATGGTAAGCGAAGCGCATCAGATAATGTTGCATTTGCCATCAGTTAATGGGAATTAGCCATGGTCC3' |
| P311 *sodC* F | 5'CTAGGTTACGACGTACCGTAAGCACTTTTAGGAATAGCCGCCGTTCAAAAGTGTAGGCTGGAGCTGCTTC3' |
| P312 *sodC* R | 5'CGCTTTCGCAACTTGAGCAAGCACCGCCGCAAGGTACGCTGGCTTCTTAAATGGGAATTAGCCATGGTCC3' |
| P334 *soxS* F | 5'ACTCCCCAACAGATGAATTAACGAACTGAACACTGAAAAGAGGCAGATTTGTGTAGGCTGGAGCTGCTTC3' |
| P335 *soxS* R | 5'CAATGGATGGGGTAATTACCCGCGCGGGAGTTAACGCGCGGGCAATAAAA ATGGGAATTAGCCATGGTCC3' |
| P336 *soxR* F | 5'CATCTGTTGGGGAGTATAATTCCTCAAGTTAACTTGAGGTAAAGCGATTT GTGTAGGCTGGAGCTGCTTC3' |
| P337 *soxR* R | 5'AGAGAGAAAGACAAAGACCGGAAAACAAACTAAAGCGCCCTTGTGGCGCT ATGGGAATTAGCCATGGTCC3' |
| **Purpose: Verification of mutants** | |
| P75 Km internal F | 5'ACAACAGACAATCGGCTGCTCTGATGC3' |
| P61 Km F | 5'GTGTAGGCTGGAGCTGCTTC3' |
| P62 Km R | 5'ATGGGAATTAGCCATGGTCC3' |
| P219 *copA* down | 5'CCGATTTTCATGCATCCTG3' |
| P323 *sodC* F | 5'TAGCGTCACCATTACTGAAAC3' |
| P324 *sodC* R | 5'CTTCATCCAGTGATTTCAGAC3' |
| P325 *sodB* F | 5'AGTATCACTACGGCAAGCAT3' |
| P326 *sodB* R | 5'TAATAAGCGTGTTCCCAGAC3' |
| P327 *sodA* F | 5'CCATCAGACTTACGTCAACA3' |
| P328 *sodA* R | 5'CAGTGGAGAGTCCTGGTTAG3' |
| P330 *sodA* down | 5'CCGTTGGCGATGGTTCATTTT3’ |
| P332 *sodB* down | 5'TGTTAACGCCTCATCCGCGTT3’ |
| P333 *sodC* down | 5'TACTGCTCGCTTTCGCAACTT3’ |
| P338 *soxR* F | 5'CTATGAAAGCAAAGGGTTGA3' |
| P339 *soxR* R | 5'CCGGTACCTTCTTCTCCTAA3' |
| P341 *soxR* down | 5'GAATGAGGTGTGTTGACGTC3’ |
| P342 *soxS* F | 5'ATCTTATCGCATGGATTGAC3' |
| P343 *soxS* R | 5'TGAGAGACATAACCCAGGTC3' |
| P345 *soxS* down | 5'GCCGCAGGTGTTTATGCAAT3’ |
| **Purpose: qPCR** | |
| P279 *sodA* F | 5'AAGAGCTGATCACCAAACTG3' |
| P280 *sodA* R | 5'TTCCAGAACAGACTGTGGTT3' |
| P281 *sodB* F | 5'GAAGAGATTATTCGCAGCTC3' |
| P282 *sodB* R | 5'CAGGCAGTTCCAGTAGAAAG3' |
| P283 *sodC* F | 5'AGCGTCACCATTACTGAAAC3' |
| P284 *sodC* R | 5'AATATGGAAGCCATGTTCAC3' |
| P285 *soxS* F | 5'ATCTTATCGCATGGATTGAC3' |
| P286 *soxS* R | 5'CGTTGCAAGTACCACTTTG3' |
| P287 *soxR* F | 5'TCTATGAAAGCAAAGGGTTG3' |
| P288 *soxR* R | 5'GACATATCGCAACACATCAC3' |
| P293 *gapA* F | 5'AAGTTGGTGTTGACGTTGTCGC3' |
| P294 *gapA* R | 5'AGCGCCTTTAACGAACATCG3' |
| P250 *copA* F | 5’GCTACTGCTTTCCCGTCAAC3’ |
| P251 *copA* R | 5’TGACCAACAGGTCGATACCA3’ |
| P181 *cusC* F | 5’AATGTCGCGCAAAGCTATTT3’ |
| P182 *cusC* R | 5’CGACAAACGCATATGACTGC3’ |
| P407 *mntH* F | 5’TCGGCAACACCAGTATGACC3’ |
| P408 *mntH* R | 5’ATTCCGCCACCAAATGGGAT3’ |
| P409 *sitA* F | 5’CGTCACACCCGAAGAGACAA3’ |
| P410 *sitA* R | 5’CAGGGGGCACAACTGATTCT3’ |

**^a^**F, forward; R, reverse; Km, kanamycin; down, downstream or 3’
