# Supplementary figures and images for: Copper primes adaptation of uropathogenic Escherichia coli to superoxide stress by activating superoxide dismutases
Source: PLoS Pathog. 2020 Aug 26;16(8):e1008856. doi: 10.1371/journal.ppat.1008856 (PMC7478841; doi:10.1371/journal.ppat.1008856)

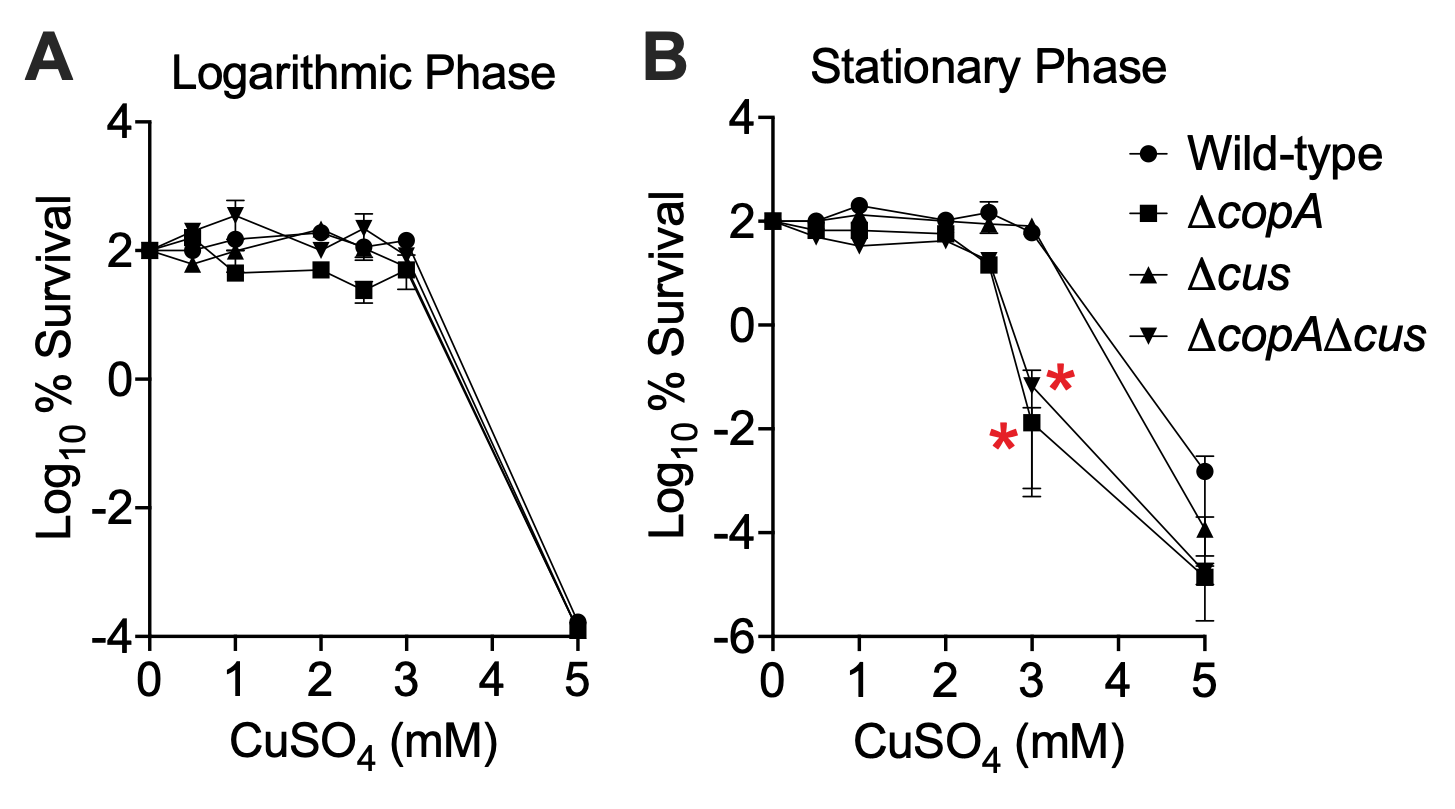

Supplement: S1 Fig — (TIF) [file ppat.1008856.s002.tif]

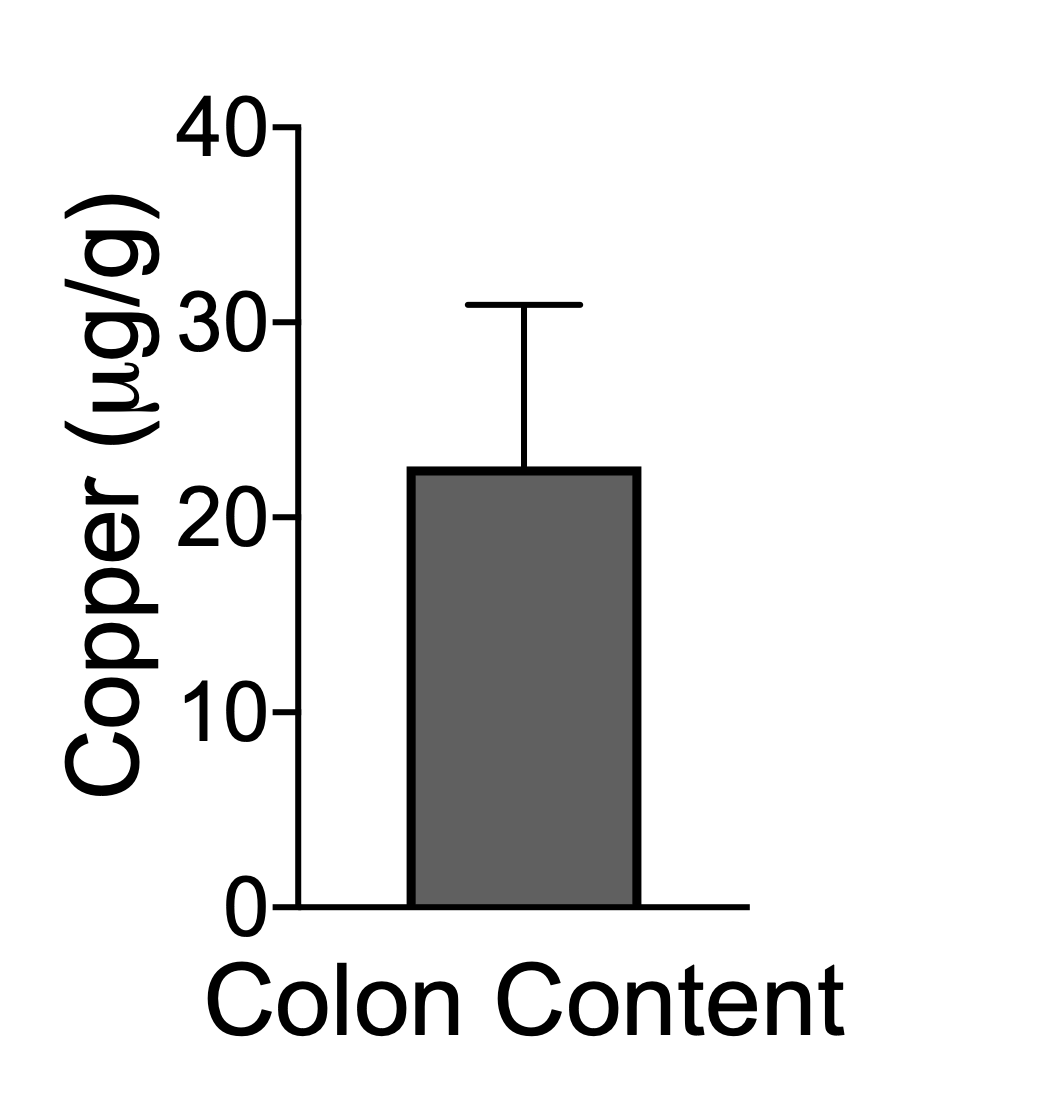

Supplement: S2 Fig — (TIF) [file ppat.1008856.s003.tif]

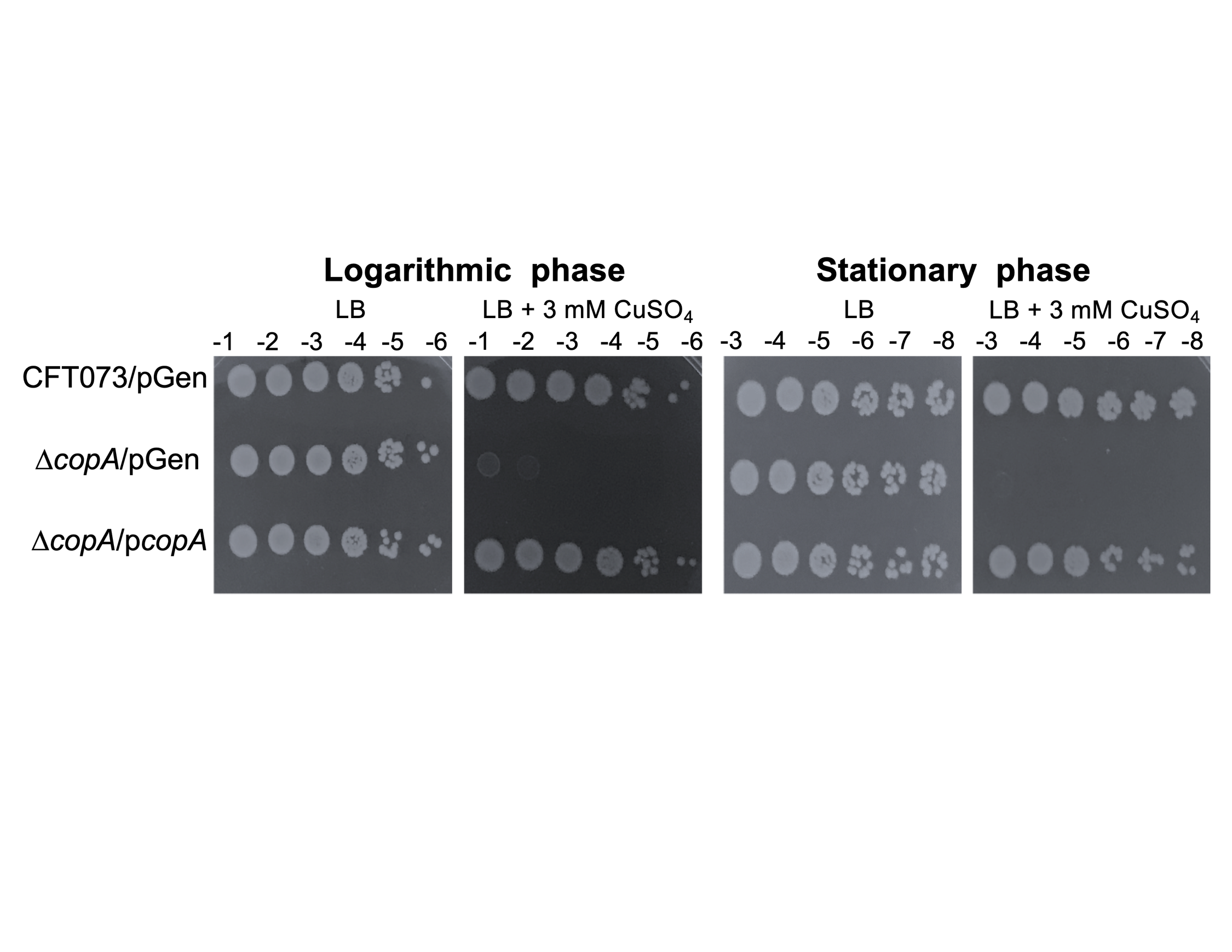

Supplement: S3 Fig — (TIF) [file ppat.1008856.s004.tif]

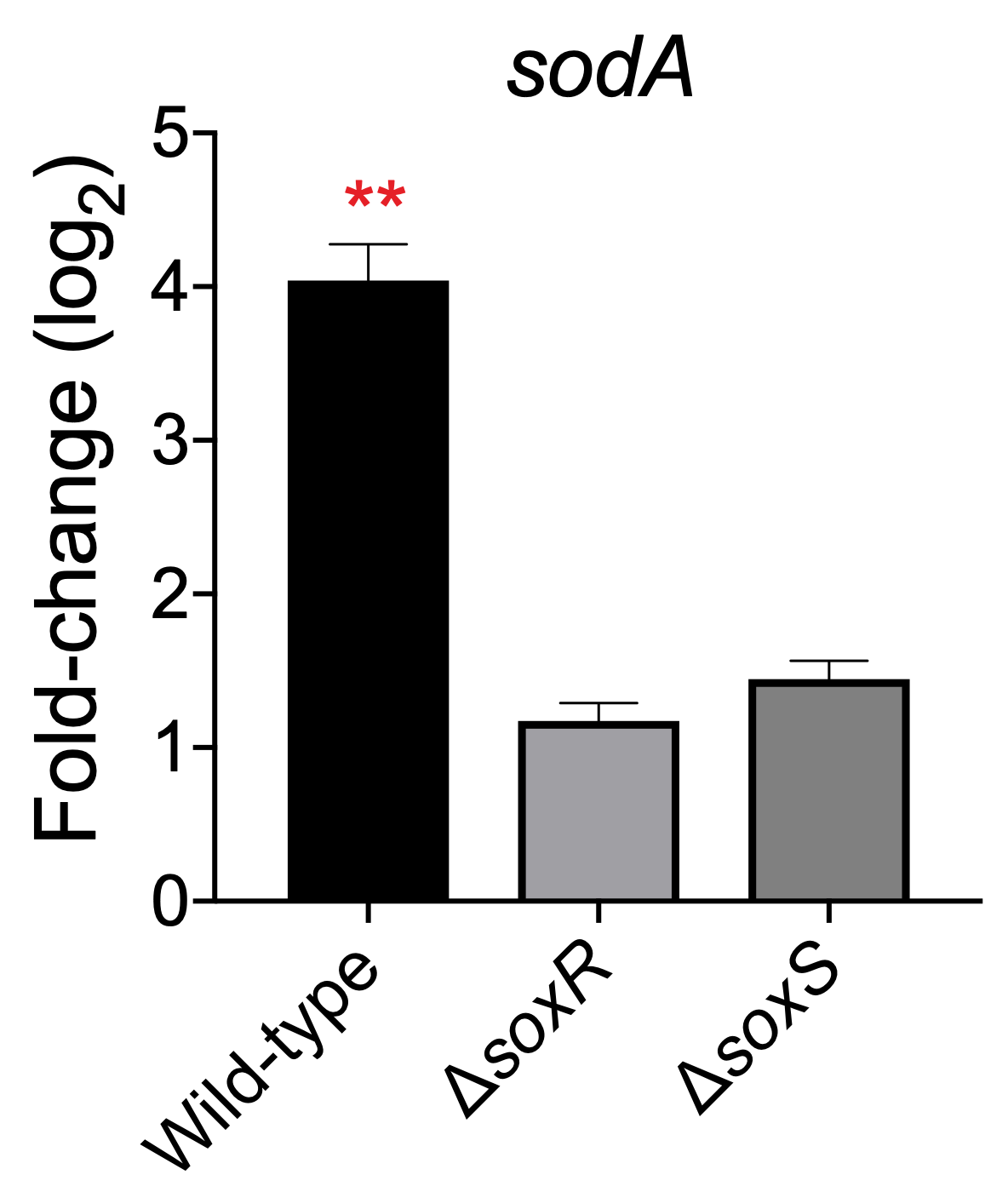

Supplement: S4 Fig — (TIF) [file ppat.1008856.s005.tif]

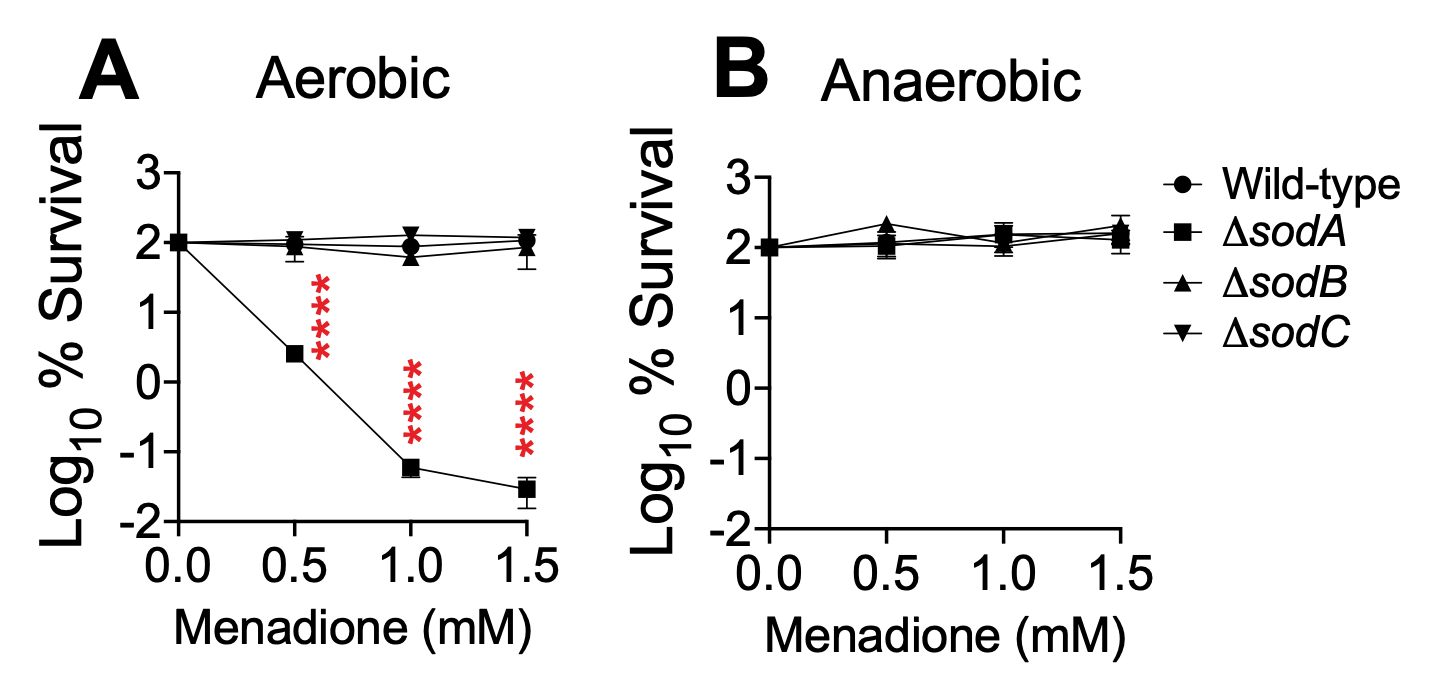

Supplement: S5 Fig — (TIF) [file ppat.1008856.s006.tif]

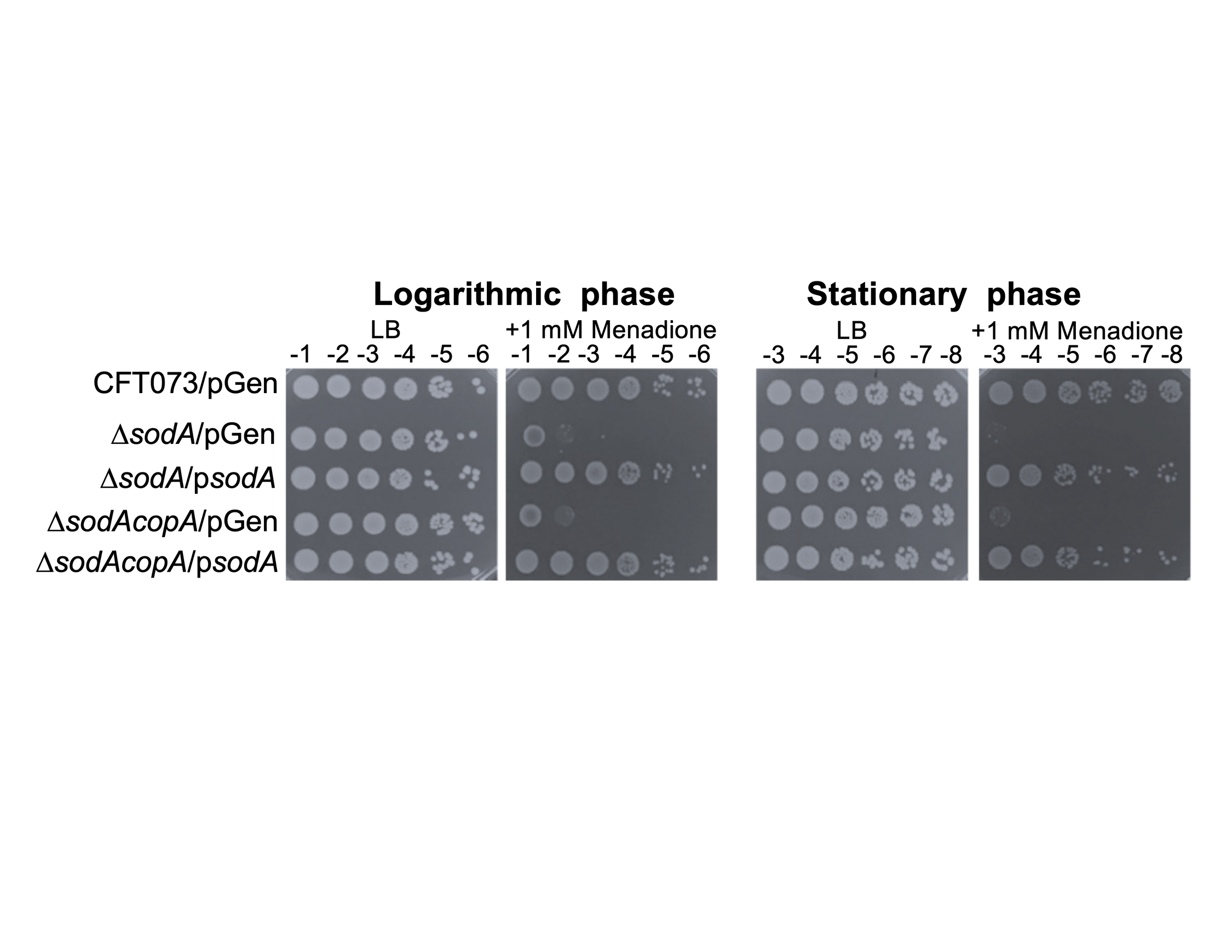

Supplement: S6 Fig — (TIF) [file ppat.1008856.s007.tif]

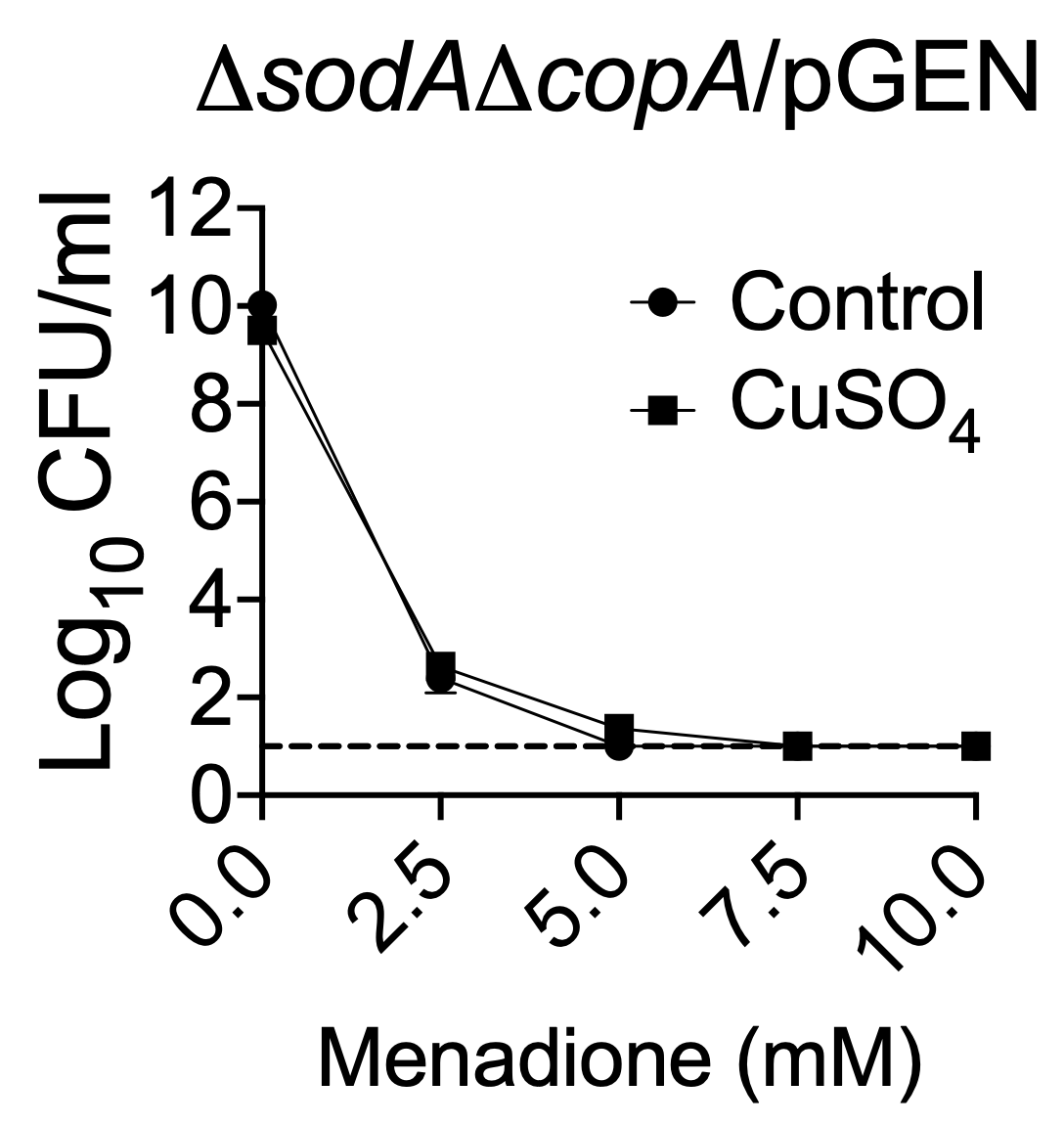

Supplement: S7 Fig — (TIF) [file ppat.1008856.s008.tif]
